# Humans Optimize Decision-Making by Delaying Decision Onset

**DOI:** 10.1371/journal.pone.0089638

**Published:** 2014-03-05

**Authors:** Tobias Teichert, Vincent P. Ferrera, Jack Grinband

**Affiliations:** 1 Department of Neuroscience, Columbia University, New York, New York, United States of America; 2 Department of Radiology, Columbia University, New York, New York, United States of America; 3 Department of Psychiatry, University of Pittsburgh, Pittsburgh, Pennsylvania, United States of America; University of California, Davis, United States of America

## Abstract

Why do humans make errors on seemingly trivial perceptual decisions? It has been shown that such errors occur in part because the decision process (evidence accumulation) is initiated before selective attention has isolated the relevant sensory information from salient distractors. Nevertheless, it is typically assumed that subjects increase accuracy by prolonging the decision process rather than delaying decision onset. To date it has not been tested whether humans can strategically delay decision onset to increase response accuracy. To address this question we measured the time course of selective attention in a motion interference task using a novel variant of the response signal paradigm. Based on these measurements we estimated time-dependent drift rate and showed that subjects should in principle be able trade speed for accuracy very effectively by delaying decision onset. Using the time-dependent estimate of drift rate we show that subjects indeed delay decision onset in addition to raising response threshold when asked to stress accuracy over speed in a free reaction version of the same motion-interference task. These findings show that decision onset is a critical aspect of the decision process that can be adjusted to effectively improve decision accuracy.

## Introduction

Humans have the remarkable ability to make fast and accurate decisions in a seemingly endless number of different tasks [1]–[3]. However, in some situations it may be unnecessary, undesirable or even counterproductive to initiate a decision. Despite intense investigation of virtually every other component of the decision process, it still not clear whether decision onset is under cognitive control, or whether decisions are initiated automatically by the presence of an appropriate sensory stimulus. Here we tested whether humans will delay the onset of a decision process to increase response accuracy in a situation when it is beneficial to ignore the initial pulse of sensory evidence that can be dominated by salient rather than task-relevant information.

**Figure 1:**
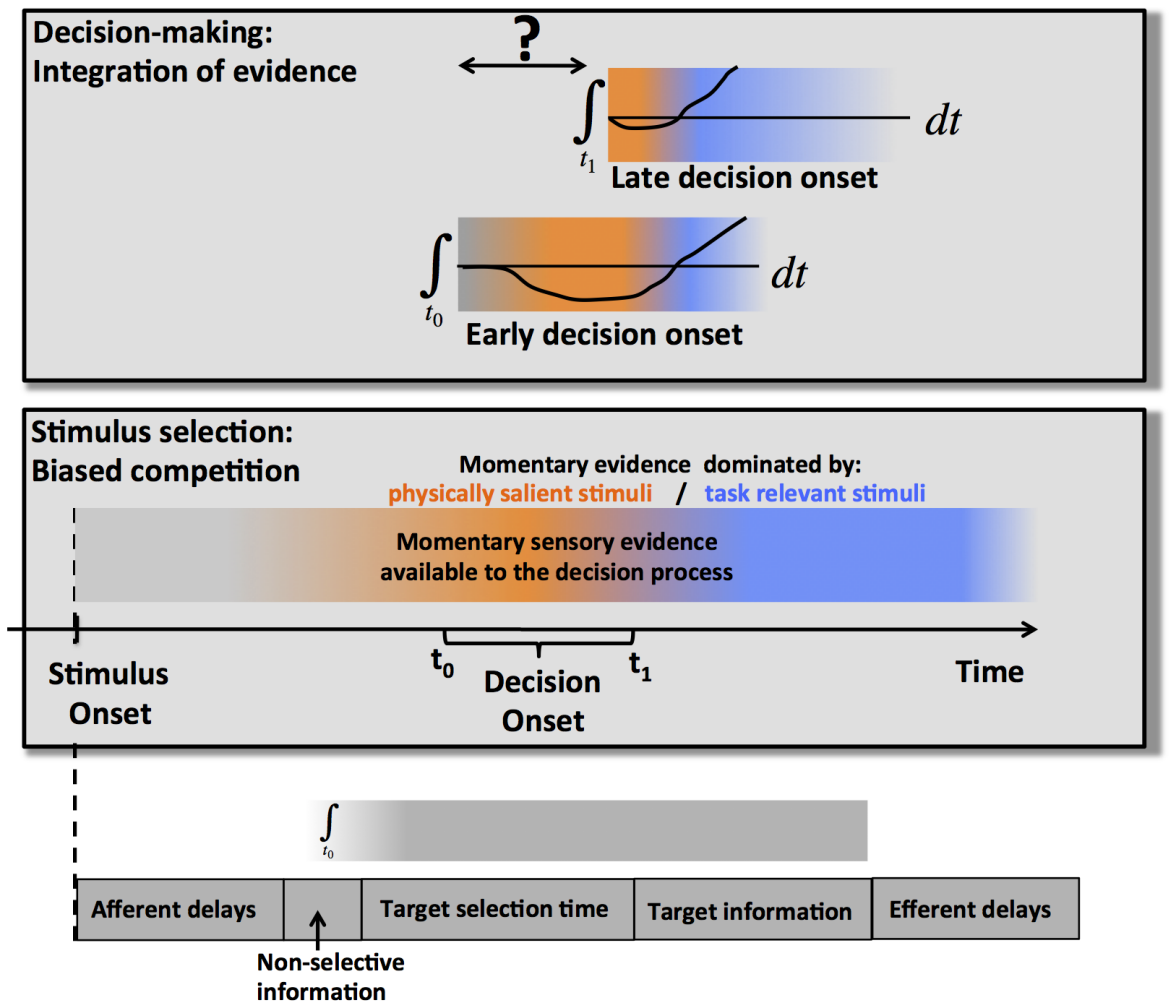
represents a simplified schematic of the problems that arise when decisions are made in the presence of salient but irrelevant distractors. The lowest panel represents a quick overview of the relevant processing stages. The time-course of the sensory representation is determined by afferent delays, the time it takes for sensory evidence to develop stimulus-selective responses, and the time it takes selective attention to extract the relevant sensory information from salient distractors. The timing of the decision-making stage is determined by the onset of the integration process and its termination. The duration of the last stage of processing is determined by the efferent delays. For the current purposes, we assume that the afferent delays as well as the delay for stimulus-selective sensory information to reach the decision stage is hard-wired and cannot be changed through cognitive control. However, we test the possibility that the onset of the decision process may be under cognitive control. The middle panel provides a more detailed look at the mix of momentary sensory information available to the decision process as a function of time: the initial phase is dominated by random internal fluctuations or the first pulse of sensory information that is not stimulus selective (grey), the second phase is dominated by physically salient stimuli (orange) and the third phase is dominated by the task-relevant stimuli, regardless of salience (blue). The time of transition from the first to the second phase is determined by afferent delays, the transition from the second to the third phase depends on how quickly selective attention can be allocated to the target. The top panel examines the effects of adjusting decision onset: if the decision process is initiated early, it will integrate information from physically salient stimuli that may or may not be relevant for the current task. If decisions are initiated late, response latencies may be prolonged unnecessarily.

Most decisions are based on a subset of stimuli that appears in close temporal and/or spatial proximity to other, task-irrelevant information. Often, the irrelevant stimuli interfere with the processing of the relevant stimuli especially when the distractors are physically salient [4]–[7] and/or are associated with behavioral responses that are similar to those of the current task [1], [2]. Thus, accurate decision-making critically depends on top-down attentional selection that enhances task-relevant and/or suppresses irrelevant information [8], [9]. The engagement of selective attention takes time [10], [11], and it has been suggested that errors occur in part because the decision process (evidence accumulation) is initiated before selective attention has isolated task-relevant sensory information from salient but irrelevant distractors [11]. Nevertheless, it has never been suggested that subjects might increase accuracy by delaying the onset of the decision process (*the onset mechanism*, Figure 1). This possibility is not precluded by the generally accepted finding that subjects trade speed for accuracy by increasing the amount of evidence that will trigger a response (*the threshold mechanism*) [12]–[15], as both mechanisms could be used in parallel.

Many studies of decision-making have presented an isolated stimulus on a uniform background. The lack of distractors minimizes the need for selective attention and allows the simplifying assumption that the rate of evidence accumulation (i.e. drift rate) stays constant over the time course of the decision. In such conditions, the onset of the decision process does not affect response accuracy and cannot be distinguished from factors that influence non-decision time. Decision onset only has an effect on accuracy if the quality of the sensory information changes over time, as may happen when selective attention gradually isolates the relevant information from among distractors. The more salient the distractor and the longer it takes to re-allocate attention to the target stimulus, the more beneficial it will be to delay decision onset.

To test whether subjects are able to delay decision onset, it is necessary to (1) use a task in which drift rate is known to change over time and (2) explicitly measure its time-course in order to predict the effects of decision onset on accuracy. While it is generally accepted that selective attention leads to changes of drift rate in Stroop-like response interference tasks [11], [16], [17], explicit quantitative measurements of the time course of selective attention within these tasks have been difficult to acquire. Without these measurements, it is not possible to quantify the effects of decision onset and test whether subjects delay decision onset to increase response accuracy.

In the current set of experiments we present two novel methods: (1) a motion interference task which requires feature-based selective attention and has a non-stationary drift rate and (2) the cyclic deadline paradigm which allows us to estimate the time-course of selective attention and the resulting changes in drift rate. This enabled us to answer two main questions regarding the role of decision onset for optimal decision-making: (1) Is delaying decision onset an effective way to increase response accuracy? (2) Do humans delay decision onset when asked to stress decision accuracy over speed? The answers will shed light on an important but rarely studied aspect of the decision process, namely its initiation. In particular, it will show whether decisions are initiated automatically by the presence of appropriate stimuli, or whether the time of decision onset can be controlled to meet task requirements.

## Methods

### Ethics Statement

All participants provided written signed informed consent after explanation of study procedures. Experiments and study protocol were approved by the Institutional Review Boards of Columbia University and New York State Psychiatric Institute.

### Experimental overview

Our goal was to determine whether decision onset is under cognitive control and used to adjust speed and accuracy. We developed a motion interference task in which two sets of moving dots were spatially superimposed. This task requires that feature-based selective attention identify the direction of motion of the target dots, while ignoring a set of more salient distractor dots. Our study was divided into three parts. First, we measured reaction times (RT) and accuracy when subjects were asked to stress either speed or accuracy (***RT paradigm***). Second, using a novel response signal paradigm (*cyclic deadline* or ***CD paradigm***, see below), we measured accuracy as a function of how much time subjects were allowed to process the stimulus before being forced to respond. Using a modified diffusion model, data from the CD paradigm allowed us to estimate the duration of the stimulus selection process as well as drift rate as function of time from stimulus onset. Third, using the time-dependent drift rate estimated from the cyclic deadline paradigm, we modeled RT and accuracy that was previously observed in the RT paradigm. Our results show that the onset mechanism can be a more effective way to trade speed for accuracy than the threshold mechanism and that subjects implement both mechanisms during perceptual decision-making.

### Experimental setup

The experiments were performed on two MacBook Pro computers, with 13 and 17-inch screens. Individual subjects were tested consistently in one of the two setups. The task was programmed and executed with Matlab2009a (www.mathworks.com) using Psychtoolbox3 (www.psychtoolbox.org). Experiments were conducted in a dark experimental room. Screen resolution was set to 1280 by 800 pixels. Viewing distance was 24 inches.

### Subjects

A total of 13 subjects performed the RT paradigm. One of the 13 subjects was an author of the study. The other 12 subjects were naïve to the purpose of the study. Six of these subjects (1 author and 5 naïve subjects) went on to the cyclic deadline paradigm. In addition, a seventh subject (another one of the authors) performed the cyclic deadline task without participating in the RT paradigm. In summary, a total of seven human subjects (1 female, 5 naïve), between 22 and 41 years old (mean: 30 years), participated in the cyclic deadline paradigm. Subjects 1 and 2 corresponded to two of the authors of the study, subjects 3-7 were naïve. A smaller number of subjects was used in the CD paradigm because (1) data collection in this task is very time-intensive and (2) there was less variability between subjects, thus reducing the need for a bigger sample. Results from the CD paradigm may be less variable because the paradigm minimized the range of available strategies and their impact on the results.

### The motion-interference task

Subjects viewed stimuli that consisted of two spatially superimposed streams of moving random dots. One stream was darker, and the other brighter than the uniform grey background [18]. Prior to stimulus onset, a luminance cue indicated which dot-stream was to be attended. The subjects indicated the direction of motion of the target dots with a button press of the left or the right index finger while ignoring the distractor dots. The distractor dots moved either in the same direction (congruent condition), the opposite direction (incongruent condition), or orthogonal to the target dots (neutral condition). For all trials, the distractor dots had higher contrast relative to background than the target dots. This ensured that the distractor motion was more salient than the target motion [19], [20], making selective attention necessary for the successful performance of the task. The task was presented to the subjects both in the context of the cyclic deadline paradigm and as a free reaction time task.

#### Stimuli

The physical parameters of the dots were set such that the task could be performed with greater than 99% accuracy in the absence of distractor dots. Luminance value of the background was set to the mean luminance of the monitor. The luminance of the target dots was either 32% above (“white” dots) or below (“black” dots) the mean luminance. Distractor dots had luminance values equal to the maximum (“white”) or minimum (“black”) luminance of the monitor. Motion coherence was set to 1.0 for both target and distractor dots. Each dot stream consisted of 50 dots. All dots had a lifetime of 30 frames. Dots were presented within a 128 pixel wide circular aperture. Any dot that moved outside the aperture was replotted within the aperture at a randomly selected position. A stationary, constantly illuminated fixation dot was presented in the center of the aperture.

#### Free reaction time (RT) paradigm

In the free reaction time version of the task (*RT paradigm*), subjects were asked to perform the motion-interference task either as quickly as possible (*speed instruction*) or as accurately as possible (*accuracy instruction*). Subjects performed 2 blocks of 162 trials each for the two speed-accuracy instructions. The order of the speed and accuracy block was randomized between subjects. It is important to note that in both conditions, subjects were free to respond at any time after stimulus onset.

#### Cyclic deadline (CD) paradigm

**Figure 2:**
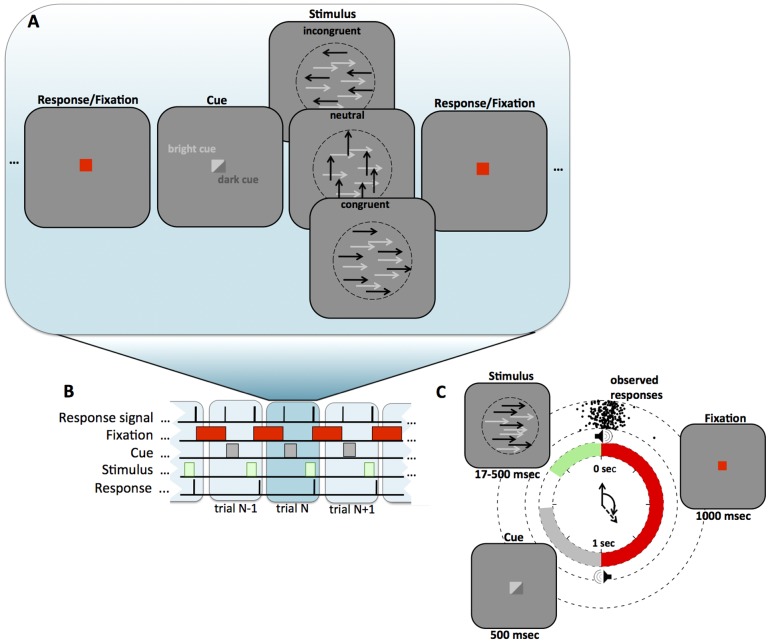
Cyclic Deadline Task. **(A)** A cue instructed subjects to attend to one of two streams of coherently moving random dots and report its direction of motion by pressing a button with their left or right index finger. The relevant stream of target dots was either lighter than the background, in which case the distractor was black, or darker than the background, in which case the distractor was white. The distractor dots could move either in the same or opposite direction as the target (congruent and incongruent condition). In addition, the distractor could move upwards, i.e. orthogonal to the axis of motion of the target dots (neutral condition). (**B/C**) The task was performed in a cyclic manner requiring a response from the subject once every 2 seconds. The time of the intended response was indicated by an auditory click. Clicks were presented once every second to convey a stronger sense of rhythm, but responses were only required on every other click, following the cue and stimulus presentation. Stimulus duration was randomized between blocks of trials.

In the cyclic deadline paradigm, trials were presented at regular intervals once every two seconds (Figure 2, see also [21]). The cyclic nature of the task was conveyed to the subjects by regular clicks that occurred once every second. The cue indicating which stimulus to attend coincided with the first click. The dot stimuli appeared at varying times prior to the second click and disappeared in time with the second click. Subjects were required to respond at the time of the second click, without regard to how long the stimulus had been on the screen. Viewing duration was manipulated between blocks of 60 trials lasting from 1 to 30 frames (16.7–500 ms).

#### Training

Prior to the RT paradigm, subjects performed one practice run to acquaint them with the task. The cyclic deadline paradigm was more challenging and required several training sessions. In the initial training sessions they learned to perform the task with very long viewing times (500 ms) until reaching a criterion of more than 95% correct responses for all trial types. Subsequent training sessions were performed with very short viewing times (≤83 ms) to accustom subjects to respond at the required deadline even when they could not acquire sufficient information to perform the task correctly. In this case, subjects were required to respond randomly with either the left or right index finger. Training continued until the standard deviation of the response was <50 ms. In the final stage of training, subjects performed the task with viewing times ranging from 16.7 to 500 ms.

### Definitions

In the cyclic deadline task, *intended processing time* was defined as the duration that subjects were allowed to process the stimulus before being required to respond. This value was identical to the duration of the stimulus on the screen. *Actual processing time* (*PT*) was defined as the time of the response minus the time of stimulus onset. Thus, PT represents the intended processing time plus trial-to-trial variability in response time. In the free reaction time version of the task, we use the term *reaction time* (*RT*) when referring to the same quantity, i.e. the time between stimulus onset and response. The use of different abbreviations is meant to emphasize whether response time was under the subject's (*RT*) or experimenter's (*PT*) control. Trials were grouped according to the direction of the distractor dots relative to the target dots (congruent, incongruent, and neutral). Trials with processing times outside a window of ±. two standard deviations around the mean processing time for the corresponding condition were excluded from the analysis.

### The biased competition model of decision-making

#### Overview

**Figure 3:**
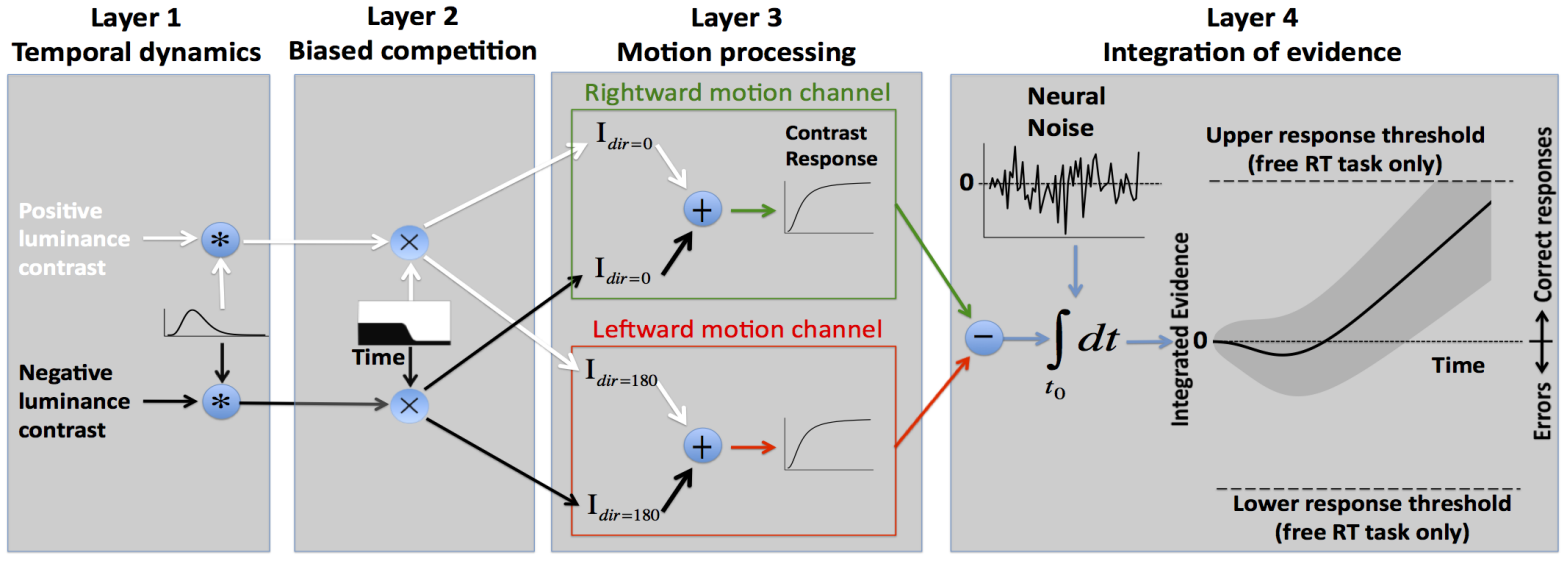
Biased competition model. The model combines two basic neural mechanisms: stimulus selection via biased competition between different luminance channels (layer 2) and the reduction of noise by integrating over time (layer 4) with a total of 6 free parameters: 3 describing biased competition, 1 describing the non-linear neural contrast response function, 1 scaling parameter, and 1 parameter describing non-decision related delays. (Layer 1) The input to the system consists of two boxcar functions that indicate the presence of the two moving random dot patterns with positive (white arrows) and negative luminance contrast (black arrows). In the first stage these inputs are filtered with a gamma-kernel to yield realistic temporal dynamics for the encoding of visual information by sensory neurons. (Layer 2) Biased competition between mutually inhibitory luminance channels is implemented as multiplicative weights *s(t)* for the target luminance and *1 - s(t)* for the distractor where 0≤*s(t)*≤*1*. Prior to stimulus onset, the weights of both luminance channels are equal (i.e. 0.5). To simulate the development of the biased competition in favor of the target luminance (black-and-white inset in layer 2), the weight of the target stimulus increases from 0.5 to an asymptote. Three parameters determine the time course of the competition: the time of the transition (τ), the speed of the transition (*σ*), and the asymptote (α). (Layer 3) The information from each luminance channel is routed to both motion channels. A stimulus elicits activity only if it matches the preferred direction of the motion channel (i.e. leftward or rightward motion). Activity from both luminance channels is summed within each motion channel. To simulate physiological neuronal responses, the activity is passed through a non-linear, Naka-Rushton contrast response function. (Layer 4) The input to the integration stage is the difference of the output of the right and leftward motion channel. In addition, neural noise is added in the form of a continuous Gaussian white noise with a standard deviation of 1 arbitrary unit per second. The percentage of correct responses as a function of time is calculated as the mass of the integrator above zero. When modeling the free reaction time version of the task, upper and lower response thresholds are included in the model. A decision is aborted and considered correct/incorrect when the diffusion process reaches the positive/negative bound. In addition, the time of integration onset t_0_ is assumed to be variable and under cognitive control.

We used a simple, physiologically inspired computational model with 6 free parameters to fit the behavioral data from the cyclic deadline paradigm (**Figure 3** and **Table 1**). After determining several key variables based on the fits to the cyclic deadline paradigm, the model was expanded to explain accuracy and RT distributions in the RT paradigm. The model consisted of rate-coding units distributed in four layers that could be grouped into two luminance and two direction-of-motion channels.

**Table 1:** List of the 10 parameters of the biased competition model of decision-making.

	Cyclic deadline paradigm	Reaction time paradigm	Valid Range	Description
Non-decision time, *ndt* [ms]	**free**	**free**	0–500	Afferent and efferent delays in the system. Difference in non-decision time between tasks/conditions were assumed to be caused by differences of efferent delays
**Onset of stimulus selection process ** ***τ*** ** [ms]**	**free**	fixed from CD paradigm	0–500	Defines when selective attention starts isolating the target stimulus
**Duration of stimulus selection process ** ***σ*** ** [ms]**	**free**	fixed/**free** (in some versions)	0–500	Defines how long selective attention needs to isolate the target stimulus
**Asymptote of stimulus selection process ** ***α***	**free**	fixed from CD paradigm	0.5–1	Defines how thoroughly selective attention will isolate the target stimulus
**Contrast response parameter ** ***I_in_***	**free**	fixed from CD paradigm	0–1	Defines the semi-saturation point of the contrast response function
Shape parameter of contrast response, *q*	**fixed/**free in one variant	fixed from CD paradigm	0.01–10.0	Defines the shape of the contrast response function. Fixed to 2 unless mentioned otherwise
Signal-to-noise ratio	**free**	fixed from cyclic task	0–100	Scalar that scales signal strength to the arbitrarily chosen noise level of 1 au^2^/sec
Starting point variability, *Var[X(t_0_)]*	0	fixed/**free** (in some versions)	0–1	The variability of the integrator unit at the time of decision onset
**Response threshold ** ***B***	NA	**free**	0–10	Threshold for response initiation – ***the threshold mechanism***
**Decision onset ** ***t_0_*** ** [ms]**	NA	**free**	0–500	Onset of integration of sensory evidence – ***the onset mechanism***

By default, six parameters were used to model the data from the CD paradigm. The top four bold-faced parameters are the core components of the model that define how drift rate changes as a function of time from stimulus onset. The lower two bold-faced parameters were introduced to model behavior in the RT paradigm. Two additional parameters (q and Var[X(t_0_)]) were allowed to vary in certain exploratory analyses.

Computational units in the input layer were selective for luminance contrast and had temporal impulse responses that converted the boxcar inputs into realistic neural dynamics. The second layer simulated biased competition between the two luminance channels. Units in the third layer were grouped into two motion channels that received inputs from both luminance channels. Information from the two luminance channels was combined within each motion channel and passed through a non-linearity. The fourth layer integrated the difference of the output of the two motion channels over time. The input to the fourth layer could be shunted by a gate that restricts the flow of information to the integrator units (temporal gating). Overall, the model is conceptually similar to other existing models of interference tasks [22] with the exception that the time course of competition between the luminance channels is directly determined by three explicit model parameters rather than indirectly through the weights and temporal dynamics of recurrent connections. Our modeling efforts are most closely related to a recent model by White and colleagues [16]. The key difference is the presence of the temporal gate that was not present in the White model. With few exceptions [23], [24], the majority of decision models do not include a gating parameter. In the particular case of decision-making under conflict, we are not aware of any model that uses temporal gating.

#### Layer 1 – temporal dynamics

The units in layer 1 received input from the two streams of dot-motion that were modeled as box-car functions representing the presence or absence of the dots on the screen. The amplitude of the boxcar was set to the absolute value of the luminance contrast. Following standard procedure [25] the visual input was smoothed by convolution with a gamma kernel. For the gamma kernel we chose a shape parameter of 7 and scale parameter of 6 ms to provide a qualitative approximation to response properties of sensory neurons in early visual cortex [26]. These values (as well as all other fixed parameter values described below) were never subjected to any optimization procedure and were selected prior to the actual data analysis.

#### Layer 2 – biased competition

Layer 2 consisted of units selective for either positive or negative luminance contrast and mediated the biased competition between them. To that aim, activity in units coding the target luminance was multiplied by the attentional selection term *s(t)* with *0<s(t)<1*; activity in units preferring the distractor luminance were multiplied by *1 - s(t)*. Whenever *s(t)* was equal to 0.5, both luminance channels were weighted equally. By setting *s(t)* to *1*, the output of layer 2 was determined exclusively by the target stimulus. This operation was used to mimic biased competition between the two luminance channels and can be thought of as a time-dependent weighting of the two luminance channels. Note that the overall sum of the two weights always added to 1. This is reminiscent of neurons in early visual cortex that respond with intermediate firing rates when two stimuli in their receptive field receive are attended to different degrees [10].

Of particular importance was the time course of the selection term *s(t)*. By default *s(t)* was set to *0.5* at the beginning of a trial, but it was allowed to increase towards an asymptote *0≤α≤1* over the time course of each trial. The transition from 0.5 to *α* was modeled by a Gaussian distribution function *Φ* that was scaled by the term *α - 0.5* and offset by *0.5*:
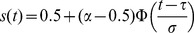
(1)


In equation (1), *τ* indicates the halfway transition point between no and full stimulus selection, and *σ* determines how fast the transition occurs. The asymptote *α* determines how completely the target stimulus is selected during the biased competition. We are certain that other functions can also be used to model the temporal dynamics of the selection process. Most likely, any sigmoidal function with three parameters (asymptote, mean, and width) would provide a reasonably good fit. Though we could have estimated the three parameters of the selection process using the single cell data of Desimone and colleagues [10], the evidence in favor of the biased competition approach would be much stronger if we were able to recover similar selection parameters directly from our behavioral data. We also experimented with asymmetric four-parameter sigmoidal functions, but were not convinced that our data allowed us to fit the fourth parameter with sufficient reliability.

#### Layer 3 – motion processing

Layer 3 consisted of two units selective for left- and rightward motion, respectively. An implicit third unit selective for upward motion was not explicitly modeled, as in our simple framework its activity would not affect the integrator unit in Layer 4 and hence, would not affect the output of the model. The units in each motion channel received input from both of the layer 2 luminance channels. The inputs were filtered with a simplistic direction-selective receptive field: if the direction of motion of the dots matched the preferred direction of the unit, its activity was multiplied by 1; otherwise it was multiplied by 0. The model could easily be expanded to include more realistic speed-tuning profiles. However, at this point we tried to keep the model as simple as possible and we do not believe that adding realistic tuning curves would change the main results and conclusions. Consequently, each stream of dots elicited activity in one of the four joint direction and contrast-selective input pathways to layer 3. The activity of the two luminance channels was summed within each motion channel. The activity within each motion channel was then divisively normalized using a Naka-Rushton function:
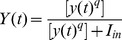
(2)


The Naka-Rushton function is a standard non-linear term used to model the cortical gain control in a variety of settings [25], [27], [28]. The exponent determines the shape of the non-linearity, typically taking a value of ∼2 [25]. To simplify the model and reduce the number of free parameters, we fixed *q* to a value of 2. In one follow-up analysis we fit *q* to the population data and recovered a value of 1.31. Using this value rather than *q = 2*, did not change the main findings regarding decision onset; thus for all remaining analyses we allowed *q* to remain fixed. The divisive inhibition term *I_in_* was a free parameter of the model. (*I_in_)^1/q^* controls the midpoint of the saturation and is also referred to as the semi-saturation point. The smaller *I_in_*, the earlier the neurons saturate their firing rate. The output of layer 3 and the effect of *I_in_* is illustrated in Figure S1.

#### Layer 4 – integration of evidence

Layer 4 consisted of a single integrator unit that received input from the two layer 3 units. The inputs from the two layer 3 units had opposite signs. The sign of the unit selective for the direction of motion of the target was arbitrarily set to +1 and vice verse. The output of the layer 4 unit was determined as integration over time of the two inputs plus a noise term:
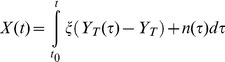
(3)


In equation (3), the subscripts 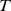
and 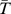
denote units with direction preference equal and opposite to the direction of motion of the target dots. The noise term *n(t)* was modeled as a white noise process with mean 0 and standard deviation of 1 au/sec. *ξ* is a scalar gain term that adjusts the amplitude of the inputs to the arbitrarily chosen variability of the noise term. *ξ* is one of the free parameters of the model. For a summary of all parameters of the model see Table 1.

Equation 3 describes a straightforward extension of the standard drift diffusion model (DDM) to situations with time-dependent drift rate. The only new component that is not present in the standard DDM is the variable *t_0,_* which refers to the time at which the integrator starts its integration process and can be thought of as a gate that regulates the flow of information to the integrator indicating when the decision process is initiated. Alternatively, *t_0_* can represent the time at which the value of the integrator is reset. This reset may be related to the dip in firing rate that can be observed in LIP neurons immediately after the onset of the relevant sensory information [29]. The effect of the reset was modeled by setting the state of the integrator at time *t_0_* equal to a Gaussian distribution with mean 0 and standard deviation sigma.

In the cyclic deadline paradigm, subjects could anticipate the onset of the relevant stimuli and were encouraged to make the most use of the available sensory information thereby allowing us to assume that they began integrating information at the earliest possible time. To model data from the cyclic deadline task, the decision onset (*t_0_*) was therefore set to the time at which stimulus selective information first reached the integrator unit. This assumption is consistent with many diffusion models that do not have an explicit gate parameter *t_0_*, but implicitly assume that integration starts as soon as information is available. Starting-point variability was set to zero to model the data from the cyclic deadline paradigm.

To model response accuracy in the cyclic deadline paradigm, we made two assumptions: (1) the decision variable *X(t)* is not bounded by a decision threshold, and (2) subjects have access to the decision variable even though the integration has not terminated at a response threshold. This was implemented by allowing the model to choose the correct response when *X(t)* was greater than zero at the time of the forced response, and the incorrect response when *X(t)* was less than zero [30], [31]. The percentage of correct responses as a function of time was calculated from the expected value and variance of *X* at time *t*:
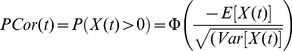
(4)where *Φ* is the distribution function of the normal distribution. The variance of *X(t)* is given as the variance of the integrated white noise process plus the variance of *X(t)* at the time *t_0_* : 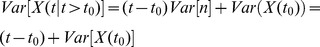
(5)


For the cyclic deadline task *Var[X(t_0_)]* was set to zero (see above). The expected value of *E[X(t)]* was calculated from equation (3) while setting the noise term *n(t)* to zero. In summary, response accuracy in the cyclic deadline task as a function of processing time was predicted with 6 free parameters. Table 1 provides a summary of all free variables. Four of the free parameters determine drift rate as a function of time during the trial. One parameter determines non-decision time and the last parameters scales stimulus strength relative to the noise amplitude that was arbitrarily set to 1 au^2^/sec.

The model was fit to the data either for each subject separately, or by combining all 7 subjects that performed the cyclic deadline task. In order to combine subjects, we first fit the model to each subject individually to estimate non-decision time. Before combining data across subjects, we subtracted individual non-decision time from processing time and added the mean non-decision time averaged across all subjects. This allowed us to combine subjects with different non-decision time.

Three general comments are in order to place these modeling efforts into context. (1) We make no claim that our model is superior to existing models when it comes to describing the temporal dynamics of the stimulus selection/drift rate over time in the cyclic deadline paradigm. Other models with a similar number of free parameters will probably provide similarly good fits. Rather, we use this specific model because its parameterization is very descriptive on the level of abstraction that is relevant for our analysis. (2) The key question that we try to answer with this model is whether or not the temporal gating parameter, i.e., decision onset, is necessary to model human behavior in the RT paradigm (see below). (3) The key parameter of interest (decision onset in the RT paradigm) is independent of the specific model that is used to estimate drift rate from the cyclic deadline paradigm.

### Modeling speed-accuracy tradeoff in the RT paradigm

The model as described above was used to predict the data from the cyclic deadline paradigm where the termination of the decision process was under experimental control. This variant of the model never terminates a decision process by itself and hence could not be used to predict reaction time distributions. In order to predict reaction time distributions in the RT paradigm, we added a mechanism that allowed the model to abort the decision process (i.e. a decision threshold or bound, *B*) based on the amount of differential evidence accumulated by the integrator unit. The decision was terminated when the decision variable *X(t)* reached a positive or a negative bound at ±*B*.

In a series of simulations, we allowed several parameters of the model to vary in order to provide a satisfactory fit to the speed and accuracy condition of the RT paradigm (for an example fit see **Figure S3**). All 4 parameters that determine drift rate as a function of time, as well as the scaling parameter ξ, were fixed to the values estimated from the cyclic deadline task. Because not all of the subjects in the RT paradigm had completed the cyclic deadline paradigm, we decided to use the parameters from the combined group-based fit, rather than the individual subject fits. The use of the group-level parameters is supported by the finding that all subjects had similar drift rate estimates.

In the RT paradigm, twelve of the subjects showed unimodal RT distributions. In eight of these subjects it was not necessary to remove any outliers. In four of these subjects, we removed a small fraction of trials (<2%) with either untypically short or long RTs. The remaining subject showed bi-modal RT distributions with a clearly defined cluster of anticipatory responses that was larger in the speed (10%) than accuracy condition (4%). These anticipatory trials were excluded for the fitting of the RT distributions.

Some parameters were either not present in the cyclic deadline model (decision bound *B*, and decision onset *t_0_*), or might take on different values in the RT paradigm (non-decision time, starting-point variability, stimulus selection time). The additional parameters in the RT paradigm had either one degree of freedom (same value for both speed and accuracy condition) or two degrees of freedom (different values for speed and accuracy condition). We initiated the fitting process using the simplest model possible, in which only Bound was allowed to vary between the speed and accuracy condition. This model provided an unsatisfactory fit to the data (data not shown) and it became clear that it was necessary to allow non-decision time to vary between the two paradigms. The resulting fits revealed that non-decision time was significantly longer in the RT paradigm. This was true for all variants of the model that we tested. A more detailed interpretation of this finding can be found in the Discussion.

### Model implementation, fitting routines, and model evaluation

All computations were discretized at a temporal resolution of 2 ms and simulated numerically using in-house software programmed with the statistical software package R (Version 2.13). Fitting was performed with the *genoud* function that combines a genetic search followed by a standard gradient descent [32]. By default, we used a limited genetic search to identify a good starting point for the gradient descent method. The limited genetic search consisted of 10 generations with 500 individuals in each population. We used the default settings of the *genoud* function regarding the proportion of different operators (Cloning: 65 individuals; Uniform Mutation, Boundary Mutation, Non-Uniform Mutation, Polytope Crossover, Simple Crossover, While Non-Uniform Mutation, Heuristic Crossover: 62 individuals each). After a burn-in period of 5 generations, a gradient descent was conducted on the best individual of each generation using a quasi-Newton method with enforced boundary conditions (L-BFGS-B). In some instances we used a more extensive optimization algorithm to test whether the limited search provides the best fit. The extensive optimization algorithm consisted of 100 generations of 1000 individuals each with a burn-in period of 50 generations. In all instances, the results from the full and limited search were numerically identical. This is evidence that the limited genetic search was sufficient to find the best possible fits. Hence, we used the limited search in all remaining fitting procedures.

The goal of the fitting procedure was to minimize a log-likelihood of the data given a particular set of free parameters. For each of the 6 RT distributions for correct responses (congruent, neutral, incongruent in the speed and accuracy condition), we measured the 10^th^, 30^th^, 50^th^, 70^th^ and 90^th^ percentiles. We then determined the probability that the model placed in each of the 6 RT bins, and calculated the log-likelihood of the data given a specific set of parameters. Due to the limited number of trials in the error condition it was not feasible to get good RT quantile estimates for the error RT distributions. Hence, we used the limits from the correct RT distributions to bin the RT distributions on error trials of the corresponding distribution. In earlier efforts, we calculated log-likelihoods using the 2 ms native temporal resolution of our model. We discontinued the use of 2 ms bins since the fits were more sensitive to outliers. However, the initial fits that were done using the 2 ms binning provided the same qualitative results as the quantile binning procedure.

We used three different approaches to assess model fits: (1) Wilkes chi-square tests for nested models, (2) Bayesian Information Criterion (BIC) for non-nested models and (3) paired t-tests to compare parameter estimates in the speed and accuracy condition.

(1) The Wilkes chi-square test for log-likelihood ratios uses a test-statistic proportional to the difference between log-likelihood of two nested models, and compares this value to a Chi-square distribution with the number of degrees of freedom equal to the difference between the two models. We used this test on each subject as well as on the population. To extend the test to the population, we summed the log-likelihood values and the differences in the number of degrees of freedom across all subjects. (2) The BIC enables the comparison of non-nested models with different degrees of freedom by penalizing the log-likelihood for the number of free parameters in the model. In our case, most comparisons of interest involved either nested models, or non-nested models with identical numbers of free parameters. Hence, the BIC was of limited use and included only because it is a commonly used measure. (3) The tests outlined above provide a measure of whether a specific parameter can help improve the fit of a model. However, they do not test if the parameter in question differs systematically across the entire population. To test this, we used population-based paired t-tests of parameter estimates from the speed and the accuracy conditions.

### Assumptions about non-decision times in the system

The data analysis and the modeling efforts critically depend on several key assumptions regarding the delays in the system. In the following we make these assumptions explicit. (1) Non-decision time comprises afferent and efferent delays. (2) Afferent delays can further be split into two components: conduction delays that model the time from stimulus presentation to the first detectable increase of activity above baseline, and the additional time it takes before neural activity becomes stimulus selective, distinguishing the different directions of motion. (3) The afferent delays are not under cognitive control and will always be constant as long as the properties of the stimulus (contrast, spatial frequency, etc) do not change. (4a) The time it takes to select the relevant sensory information via feature-based selective attention has a physiologically defined lower limit, and subjects achieve this lower limit in the cyclic deadline paradigm. (4b) Stimulus selection time is constant in all paradigms and across speed-accuracy instructions. With the exception of assumption 4b, all assumptions are necessary for our interpretation of the data. In one of the follow-up analyses we relaxed assumption 4b to test if this can provide a better fit to the data.

## Results

### Speed-accuracy instructions in the RT paradigm

In Experiment 1, subjects performed four blocks of the RT version of the motion-interference task in which they were required to stress either speed or accuracy. In line with the instructions, accuracy and RTs were significantly higher in the accuracy compared to the speed condition (Acc: 98±1%; Spd: 90±6%, mean±std; one-sided paired t-test, df = 12, t = 25.7, p<10^−11^; Acc: 593±87 ms; Spd: 498±66 ms; one-sided paired t-test, df = 12, t = 8.2, p<10^−5^). The aim of the current study was to model both accuracy and the RT distributions in order to understand the contribution of the threshold and onset mechanisms to the observed speed-accuracy tradeoff. In line with previous studies [16], [17] we used a modified version of the standard diffusion model that allows systematic variations of drift rate over the time course of an individual trial. There are two main differences that distinguish our approach from previous ones. (1) We added a temporal gating parameter to the modified diffusion model that prevents the integration of evidence until a particular predetermined time *t_0_*. This allows us to test the role of decision onset in speed-accuracy tradeoff. (2) We set out to explicitly measure the time course of drift rate using an independent experiment (see below) rather than fitting the time course post-hoc to the data from the RT paradigm itself. These independent measurements of drift rate from the response signal task form the basis of our modified version of the standard diffusion model that, if our assumptions and measurements are accurate, should provide a plausible fit to the data in the RT paradigm. This approach of using two independent experimental paradigms was recently pioneered by Ratcliff [33] and, albeit challenging, is arguably the most informative way to analyze reaction time data. So far, however, it has not been applied successfully to understand decision-making under conflict. The following sections describe the response signal experiment that uses our novel cyclic-deadline paradigm to measure the time course of drift rate over the course of an individual trial. After that, we will return to the modeling of the data from the RT paradigm with the new modified diffusion model.

### Evaluating the cyclic deadline paradigm

**Figure 4:**
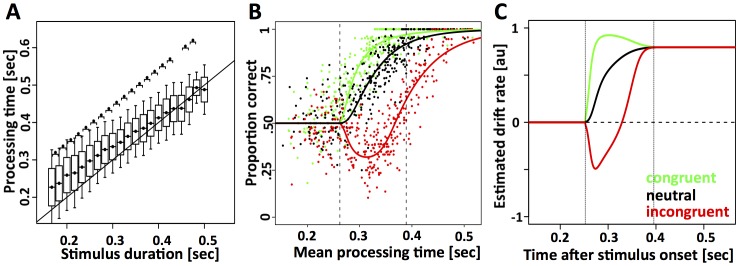
Processing times of all subjects as a function of stimulus duration, i.e. intended processing time in (A). The box and whiskers indicate mean ±one and two standard deviations, respectively. The brackets over two adjacent stimulus durations indicate significant differences at p<0.01. An additional asterisk above the bracket indicates a p-value below 0.001. In nearly all cases, a 16.7 ms increase in intended processing time resulted in a significant difference in observed processing time. **(B)** Percent correct responses are plotted as a function of mean processing time and stimulus congruency. Each dot describes a block of trials with a particular intended processing time and congruency (green-congruent; black-neutral; red-incongruent). The x-value of the dots corresponds to the mean observed processing time and the y-value corresponds to the mean proportion of correct responses. In the congruent and neutral conditions response accuracy increases monotonically as a function of RT. For the incongruent condition, in contrast, there is an initial dip with accuracy decreasing significantly below chance. The solid lines correspond to the maximum-likelihood fit of the data with the biased competition model. The left-hand vertical dotted lines correspond to the total non-decision times in the system. The right-hand dotted vertical line corresponds to the time at which the stimulus selection process has finished. **(C)** Time-resolved estimates of drift rate that give rise to the model predictions in (B). For all three conditions, drift rate converges to the same asymptote. This feature is not hard-coded into the model, but arises from the fit to the data. It indicates that attention selects the target stimulus in a winner-take-all fashion (α = 1), such that in the steady state, the identity of the distractor no longer affects drift rate. Drift rate in (C) is determined by four parameters of the biased-competition model.

In Experiment 2, subjects were required to respond at the anticipated time of the auditory response cue without regard to when or what kind of stimulus was presented. The cyclic deadline (**CD**) paradigm reliably induced significant differences in processing time (see Methods) between conditions that differed in duration by as little as 16.7 ms (**Figure 4A**). To further quantify how well subjects were able to follow the timing requirements of the cyclic deadline task we measured (1) variability of processing times around the mean (random errors), and (2) systematic deviations of the mean from the intended processing time (systematic errors). Random error was calculated as the standard deviation of single trial processing times after subtracting the mean for each block and stimulus condition. The smaller the standard deviation, the tighter the processing times are distributed around the mean. The results of this analysis are summarized in Table 2. Seven of nine subjects that were trained in the cyclic deadline paradigm had random errors that met our criterion of 50 ms or less. Based on this analysis, two subjects were excluded from participation in the main cyclic deadline experiment because they failed to meet the criterion during the training phase.

**Table 2:** Timing accuracy in the cyclic deadline task.

	Subj 1	Subj 2	Subj 3	Subj 4	Subj 5	Subj 6	Subj 7
**sd (residual PT) [ms]**	27	34	44	41	40	43	48

Timing accuracy in the cyclic deadline task measured as standard deviation of single trial processing time from the mean processing time in each block and condition.

To quantify systematic errors, mean processing times were calculated for each block of trials and distractor congruency. For each subject we modeled mean observed processing time as a function of block number – a proxy for intended processing time that was varied in blocks – and congruency. The results indicate that between 84 and 98% of the variability in mean processing time was explained by intended processing time, and hence under experimental control (see Table 3). In six of seven cases, less than 1% of the variability was due to congruency of the stimuli. Subjects 6 and 7 exhibited the weakest timing performance (see also **Figure S2**). However, the quantitative results are comparable to the other subjects. This can be taken as evidence that moderate violations from the instructions still provide interpretable results. We re-ran the main analyses excluding these two subjects. This led to small quantitative, but no qualitative, differences (see below).

**Table 3:** Determinants of processing time.

	Subj 1	Subj 2	Subj 3	Subj 4	Subj 5	Subj 6	Subj 7
**Intended PT**	97.39 (***)	96.65 (***)	88.53 (***)	91.96 (***)	92.54 (***)	86.38 (***)	84.83 (***)
**Congruency**	0.07 (*ns*)	0.29 (**)	0.52 (**)	0.15 (*ns*)	0.10 (*ns*)	0.97 (**)	1.10 (**)
**Interaction**	0.03 (*ns*)	0.22 (**)	0.15 (*ns*)	0.14 (*ns*)	0.09 (*ns*)	0.28 (**)	0.83 (*)

Percent variance of mean processing times explained by the experimental manipulations (intended processing time, congruency, and their interaction). Asterisks in brackets indicate the significance levels: (*): p<0.05, (**): p<0.01, (***):p<10^−10^.

In RT paradigms, accuracy and reaction time typically increase after error responses [34]. A somewhat more complex pattern is observed after incongruent trials: accuracy and reaction time differences between congruent and incongruent trials are reduced [17]. Changes in reaction time and accuracy can be explained at least in part as adjustments in cognitive control that change the threshold for triggering a motor response. The aim of the CD paradigm was to force subjects to respond at a particular point in time, rendering their behavior independent of such adjustments. To test independence, we fit a linear model to the accuracy and processing times for each subject as functions of intended processing time and congruency of the current trial, as well as congruency and accuracy of the previous trial (Table 4 and 5). Tables 4 summarizes the effects of accuracy and response conflict in the previous trial on processing time in the current trial. We observed a significant increase in processing time after error trials for only one of the seven subjects. On the population level there is no systematic increase in processing time after error trials (t-test, df = 6, t = −1.34, p = 0.88).

**Table 4:** Trial history effects: processing time.

	Subj 1	Subj 2	Subj 3	Subj 4	Subj 5	Subj 6	Subj 7
**Effect of previous trial error on processing time [ms]**	**2.7** (***)	−1.5 (*ns*)	−3.7 (***)	−2.1 (*)	−3.5 (***)	−10.7 (***)	1.6 (*ns*)
**Effect of previous trial response conflict on processing time [ms]**	−0.02 (*ns*)	−0.30 (*ns*)	−3.51 (***)	0.80 (*ns*)	0.95 (*ns*)	−**4.48 ** (** **)**	2.96 (*ns*)

Top row: Effect of previous trial error on current trial processing time in ms. Positive values indicate slower responses after an error trial, and vice verse. Bottom row: Effect of previous trial response conflict on current trial processing time. Positive values indicate slower responses after an incongruent compared to a congruent trial.

**Table 5:** Trial history effects: accuracy.

	Subj 1	Subj 2	Subj 3	Subj 4	Subj 5	Subj 6	Subj 7
**Effect of previous trial error on accuracy [au]**	−**0.172** (**)	**0.034** (**)	**0.098** (**)	0.006 (*ns*)	−0.020 (*ns*)	0.055 (*ns*)	0.031 (*ns*)
**Effect of previous trial response conflict on accuracy[au]**	−0.023 (*ns*)	−0.034 (*ns*)	**0.168** (*)	−0.010 (*ns*)	−0.095 (*ns*)	0.079 (*ns*)	−0.047 (*ns*)

Top row: Effect of previous trial error on current trial accuracy. Positive values indicate higher accuracy after an error trial and vice verse. Bottom row: Effect of previous trial response conflict on current trial accuracy. Positive values indicate higher accuracy after an incongruent compared to a congruent trial.

Similarly, processing times were not significantly affected by response conflict on the previous trial (t-test, df = 6, t = −0.56, p = 0.71). Tables 5 summarizes the effects of accuracy and response conflict in the previous trial on accuracy in the current trial. We observed a significant increase in accuracy after error trials for two of seven subjects. In one subject we found a significant decrease in accuracy. On the population level, we failed to find a significant increase in accuracy as a function of accuracy on the previous trial (t-test, t = 0.18, df = 6, p = 0.43). Similarly, we failed to find a significant increase of accuracy as a function of response conflict on the previous trial (t-test, t = 0.17, df = 6, p = 0.44). In summary, neither congruency nor error on the previous trial had significant effects on accuracy or processing time, consistent with the idea that the cyclic deadline task neutralizes serial order effects.

### Cyclic deadline paradigm – measuring and modeling response accuracy

Having established that the subjects perform the timing requirements of the cyclic deadline paradigm, we analyzed their choice behavior. Figure 4B shows the proportion of correct responses as a function of normalized mean processing time and distractor congruency for all subjects. As expected [11], accuracy on incongruent trials dropped below chance for medium processing times. This effect began to reverse at ∼330 ms, suggesting that momentary sensory evidence tipped in favor of the less salient target stimulus.

To quantitatively relate these findings to the dynamics of selective attention, we developed a simple computational framework that we refer to as the *biased competition model of decision-making* (**Figure 3** and **Table 1**). The model combines two well-established neural principles: stimulus selection via biased competition in sensory cortex [10] and decision making as the integration of noisy sensory information over time [30]. The model assumes that decisions are based on a time-continuous stochastic process with a starting point of zero and non-stationary Gaussian increments. The mean of the increments, i.e. the drift rate, varies as a function of distractor congruency and the ongoing dynamics of the attentional selection process. The proportion of correct responses as a function of processing time and distractor congruency is predicted by the mass of the stochastic process above zero [30], [31]. Three key parameters of the model determine how strongly attention selects the target over the distractor as a function of time from stimulus onset (**Figure 3**, black-and-white inset in layer 2). The fourth parameter of the model determines the saturation point of the non-linear contrast response function (**Figure 3**, inset layer 3, **Figure S1**). In combination with the stimulus parameters, i.e. contrast and direction of motion of the target/distractor, these four variables determine the precise time course of drift rate for a given trial. A fifth parameter scales drift rate to the amplitude of the white-noise process with an arbitrarily chosen variance of 1 au^2^/sec. The sixth parameter models all efferent and afferent non-decision related delays of the system.

Using a maximum-likelihood method we fit 6 free parameters of the biased competition model (Table 1) to the data either separately for each subject (**Figure S2**) or pooled over all subjects (**Figure 4B**). The fits not only capture the qualitative properties of the data, in particular the initial dip in the incongruent condition, but also provide an excellent quantitative approximation. Our simulations indicated that attention finalizes the selection of the target in a winner-take-all fashion approximately 150 ms after the onset of the decision process (Table 6). This suggests that systematic errors observed in the incongruent condition are caused by evidence accumulated during the first 150 ms of the decision process, allowing the possibility that delaying decision onset can improve response accuracy. In a follow-up analysis we re-ran the population fit while excluding subjects 6 and 7 that exhibited the weakest timing performance. The fits were similar with the exception of small quantitative differences. In particular, stimulus selection times were markedly shorter (∼120 ms rather than ∼150 ms when all 7 subjects were included). Using the population fit based on the 5 best subjects rather than all seven subjects did not change the main conclusions regarding decision onset in the CD paradigm.

**Table 6:** Model fit of the biased competition model to the cyclic deadline task.

	Subj 1	Subj 2	Subj 3	Subj 4	Subj 5	Subj 6	Subj 7	Mean of Fits	Subj 1-7 combined
**Non-decision time [ms]**	277	259	272	253	255	239	254	259±13	252.5
**Contrast response parameter I_in_**	0.149	0.156	0.256	0.086	0.094	0.293	0.057	0.156±0.089	0.159
**Onset of stimulus selection process [ms]**	0	0	0	43	4	56	44	21±25	0
**Duration of stimulus selection process [ms]**	113	138	124	62	118	62	76	105±30	149
**Asymptote of stimulus selection process**	1.0	1.0	1.0	1.0	1.0	1.0	0.97	1±0.01	1
**Signal-to-noise ratio**	7.34	7.98	8.13	5.13	5.82	9.93	6.99	7.33±1.58	6.57

The first seven columns show the results of the individual subjects. The column entitled “mean” presents the mean ± standard deviation of the values averaged over all seven subjects. The last column depicts the results on a population level. To that aim, all experimental blocks from subjects 1−7 were pooled after correcting processing times for individual differences in non-decision time.

Additional analyses showed that the data from the CD paradigm can also be explained if perfect integration is replaced with leaky integration. However, the main conclusions regarding the duration and selectivity of the stimulus selection process were not affected. Similarly, it is likely that other network architectures building on race processes [15] or leaky competing accumulation [31] may be able to predict the data from the cyclic deadline task. Hence, it is not our intention to make strong claims that the proposed model is the only one that can be used to fit the data. Rather, we conclude that it is one model that can capture the temporal dynamics of drift rate with sufficient quantitative detail and a very low number of free parameters that explicitly map onto core concepts such as the duration of the stimulus selection process. However, for the purposes of the current study, the scope of models under consideration was limited to perfect integration of differential evidence.

### RT paradigm – modeling the speed-accuracy tradeoff

To understand whether humans can use the onset and/or threshold mechanism to trade speed for accuracy when free to respond at any time, we extended the 6-parameter biased competition model to include a response threshold in the form of two absorbing boundaries (+B and –B) and a decision onset (t_0_). The model terminates a decision as soon as the stochastic process reaches one of the two boundaries and is considered a correct response if it reaches the positive boundary. These boundaries allowed the model to terminate a decision process and generate RT distributions for correct and error trials that can be used to fit the observed data. By varying decision onset, the model can actively choose when to initiate the evidence accumulation process.

In theory, decision onset could be controlled online, on a trial-by-trial basis depending on the presence of stimulus and/or response interference (***interference-dependent gating***). Alternatively, decision onset can be predetermined such that integration starts at a fixed point in time relative to the onset of the stimulus. The delay between stimulus onset and integration onset could be predetermined, for example, based on task requirements that stress either speed or accuracy (***task set-dependent gating***). *Task set-dependent gating* would operate in the form of a speed-accuracy tradeoff: delaying decision onset will increase accuracy (mainly for incongruent trials) while increasing response latencies for all three trial types. *Interference-dependent gating*, in contrast, would delay decision onset only when necessary (incongruent trials) and would not cause increased reaction times for the other trial types. However, the implementation of an interference-dependent gating mechanism would be costly, if at all possible, and add another layer of complexity to the decision process. The fact that the data in the cyclic-deadline paradigm could be fit with a model that assumes identical decision onset regardless of stimulus congruence, is strong evidence against interference-dependent gating. Based on the above considerations, we allowed decision onset to vary as a function of speed-accuracy instruction in the same way as response threshold.

### Task-set dependent temporal gating

**Figure 5:**
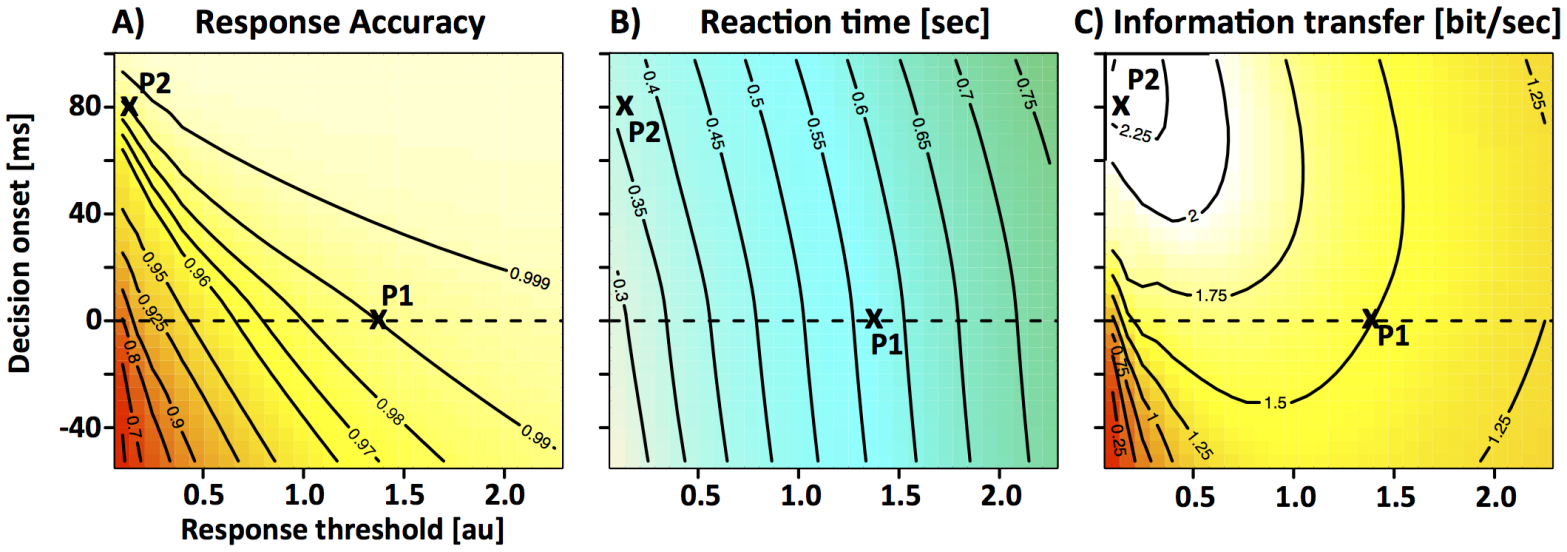
Properties of the onset mechanism. In the biased competition model, speed and accuracy can be manipulated in two independent ways — adjusting response threshold and/or decision onset. The simulations are based on the fit of the biased competition model to the data of the CD paradigm combined across all 7 subjects (**Figure 3**). Simulated response accuracy (**A**), response latency (**B**) and rate of information transfer (**C**) are depicted as functions of response threshold on the x-axis and onset of the decision process on the y-axis. The dotted line at 0 ms in each panel indicates the average non-decision time across all subjects. Our null-hypothesis states that decision onset is fixed and coincides with this time-point. Delaying decision onset increases response accuracy and RT. Increasing response threshold has the same effects, but the effect on response accuracy is weaker while the effect on RT is stronger. P1 denotes the threshold that is necessary to achieve 99% response accuracy if decision onset is fixed at 0 ms. P2 denotes a second set of parameters that leads to the same accuracy of 99% but allows decision onset to deviate from 0. Note that P1 is associated with mean reaction times around 570 ms. In contrast, P2 achieves the same accuracy with a mean RT of 370 ms. This shows that decision onset can be a very effective way to trade speed for accuracy.

In the following we tested whether task-set dependent gating, i.e., the onset mechanism, can be a more effective way to trade speed for accuracy. Based on the estimates of time-dependent drift-rate that was measured in the CD paradigm (**Figure 4C**), we simulated mean RT and accuracy as a function of the response threshold and decision onset. This allowed us to estimate the benefit of both mechanisms for the very specific task in question. In the presence of certain task requirements (e.g. “*99% correct responses*”), it is possible to define the optimal decision strategy as the strategy that achieves 99% correct responses while minimizing mean reaction time. Based on this definition, the implementation of an optimal decision strategy typically involves changes of decision onset (**Figure 5**). For example, our model predicts that an average response accuracy of 99% can be achieved by setting response threshold to ∼1.4. This setting leads to mean reaction times around 570 ms. However, if we delay decision onset by ∼80 ms the model can achieve the same accuracy for average mean reaction times around 370 ms. Note that decision onset is always reported relative to the time at which the first stimulus-selective information is believed to reach the integrator stage. Overall, the two mechanisms exhibit large and systematic differences over a wide range of the sampled parameter space (**Figure 5**): for a given increase in response latency, delaying decision onset leads to larger improvements in accuracy than raising response threshold.

In the absence of any task requirements, a strategy can be defined as optimal if it provides the highest rate of information transfer measured in bits per second. We chose *bits per second* rather than other possible metrics such as *reward per unit time* because the latter is subjective and depends on current task demands, i.e. how valuable/costly a correct/wrong response is assumed to be. The benefit of delaying decision onset becomes particularly obvious when analyzing speed and accuracy in terms of information transfer (**Figure 5C**). High rates of information transfer above 2 bits/sec can only be found for a low response threshold (<0.7) and late decision onset (>60 ms). Our simulations also show that the threshold mechanism by itself is not a very effective way to trade speed for accuracy in the current task. If the decision onset is always initiated at time 0, information transfer reaches only very moderate levels of 1.25 even if threshold is increased to around 2.1. The low rate is mainly due to the high costs in terms of RT that are incurred when increasing response threshold.

Note that this finding is in clear contrast to situations with constant drift rate. In such situations, the threshold mechanism has long been shown to be the optimal way to trade speed for accuracy. However, in conditions with changing drift rate that can be encountered for example in interference tasks, delaying decision onset would be a more effective way to trade speed for accuracy. In the following we tested whether subjects make use of the onset mechanism, or if their behavior can be explained by a suboptimal implementation of the threshold mechanism.

To test the role of decision onset and response threshold to the speed-accuracy tradeoff, we fit a series of models to the data of each subject. Except for the implementation of task set-dependent gating, we used standard drift-diffusion models with variable drift rate that was determined by modeling the data in the CD paradigm (**Figure 4C**). To that aim we fixed five of the six variables to the values obtained from the CD paradigm. Non-decision time, though estimated in the CD paradigm, was allowed to be a free parameter for two reasons. First, our simulations revealed that non-decision times were significantly longer in the RT compared to the CD paradigm (for a detailed interpretation of this finding see Discussion). Second, we allowed non-decision time to vary between speed-accuracy instructions, because it is possible that motor execution times (efferent delays) differ when subjects stress either speed or accuracy. Note that all of our models assume that afferent delays are outside of cognitive control and hence independent of speed-accuracy instructions. In addition to threshold, decision onset, and non-decision time, we allowed stimulus selection time and starting-point variability of the integrator unit to vary between speed-accuracy instructions to test alternative accounts of our data.

### Threshold and Non-decision time model (TN_22)

**Figure 6:**
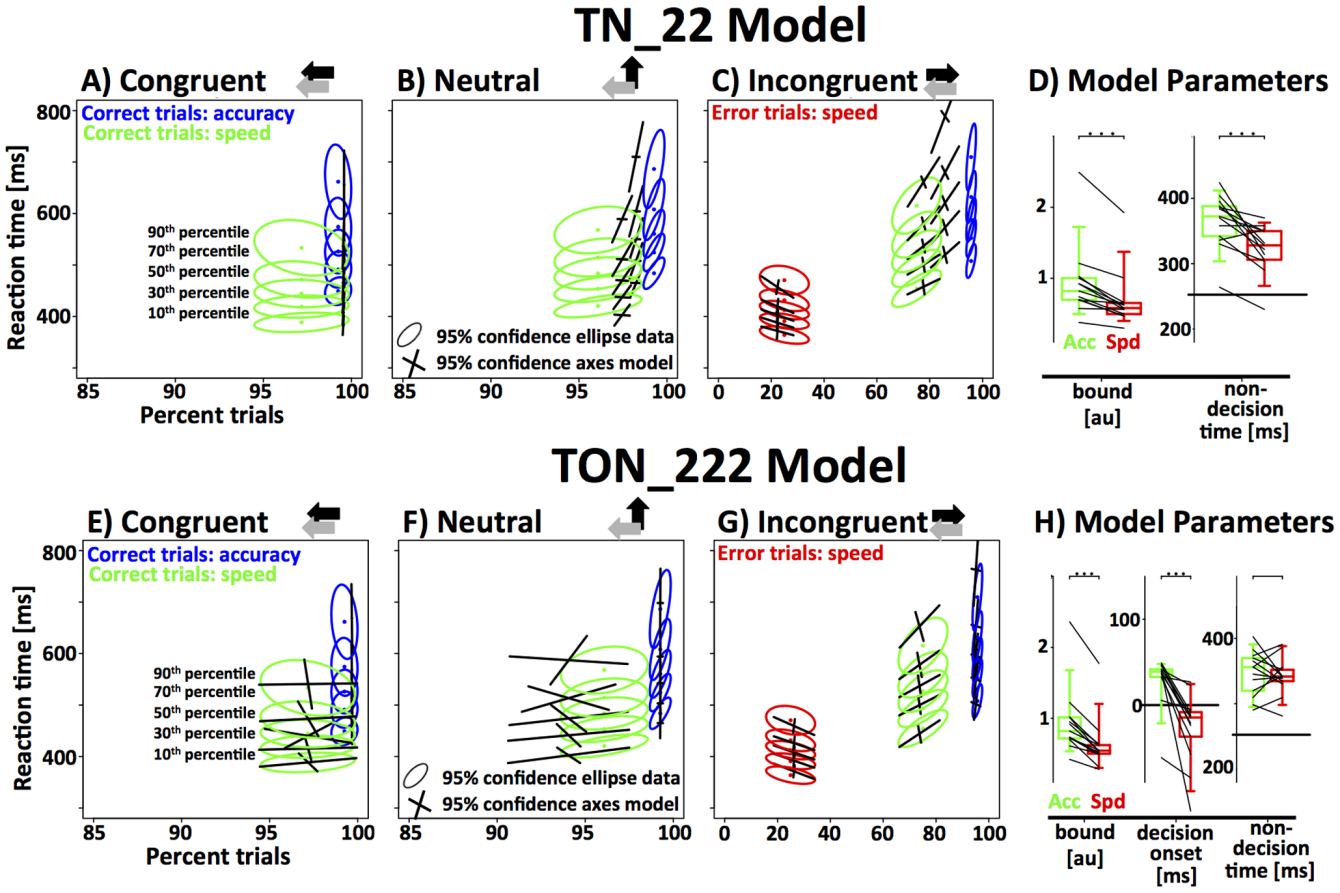
Fit of the threshold and non-decision time (TN_22) model to the data from the RT paradigm in (A–D). The first three panels represent RT distributions as a function of accuracy from the congruent, neutral and incongruent condition. Note that the x-axis covers different ranges in the incongruent panel in order to include the error trials. The empirical results are represented as 95% confidence ellipses around 5 different RT quantiles as a function of accuracy (green/red: correct/error trials speed instruction, blue: correct trials accuracy instruction). The model fit for the same quantiles is represented as the axes of the 95% confidence intervals. The rightmost panel displays the model parameters (green: accuracy instruction; red: speed instruction). Data-points from each subject are connected by a line. Results of a paired t-test are indicated above the data (bracket: ns; one star: p<0.05; two stars: p<0.01; three starts: p<0.001). The current model used 4 parameters (bound and non-decision time two parameters each) to fit the data. Note that the model fails to capture some key properties of the data. **(E–H)** Fit of the threshold, decision-onset and non-decision time (TON_222) model to the data from the RT paradigm. Conventions as in panels A–D. Note that the model provides a much better fit to the data. Speed-accuracy tradeoff is mediated through response threshold and decision onset. There are no systematic differences of non-decision time between the two conditions.

The simplest model that we will discuss in detail included 2 parameters: response threshold, and non-decision time with two degrees of freedom each. As a shorthand we refer to this model as TN_22. The letters are abbreviations of the two variables (**T**hreshold and **N**on-decision time) and the number refer to the degrees of freedom for each of the two variables. **Figure 6A–D** displays the fits of the TN_22 model to the RT distributions and accuracy in the speed and accuracy version of the RT paradigm. Because of the limited number of errors, we display the fits to the error RT distribution only for incongruent trials of the speed block (though all of the error distributions were used for the fitting process). One of the 13 subjects had substantially longer RTs and higher accuracy, an effect that was consistently captured by all variants of the model as a higher response threshold (**Figure 6D**). The quality of the fits for this subject were similar to those of other subjects. However, the inclusion of this subject increased the radii of the confidence ellipses (**Figure 6A–C**) to a point where, due to overlap, they were no longer visually informative. Hence, for visualization purposes only, we excluded this subject from the data that generated the confidence ellipses. Note that the estimated model parameters for this subject were included in all analyses.

Overall, the TN_22 model provides a good approximation to the data. We dropped individual degrees of freedom from the model to test if all four parameters are necessary (drop-one routine). The resulting sub-models are TN_21 and TN_12. Using a test for nested models, we show that removing one degree of freedom from threshold significantly decreases the accuracy of the fit (Population-based Wilkes Chi-square test for log-likelihood of nested models, df = 13, *Χ^2^ = 184*, p<10^−10^). Using the same test on the single subject level, 9 out of 13 subject showed a significant effect at p<0.01. Similarly, we show that the second degree of freedom for non-decision time is necessary (df = 13, *Χ^2^* = 246, p<10^−10^, individual subjects: p<0.01 for 9/13).

**Figure 6D** shows the parameter estimates for threshold and non-decision time as a function of speed-accuracy instruction. As expected, response threshold was significantly higher for the accuracy condition (acc: 0.94±0.51 ms, spd: 0.69±0.41 ms; paired t-test, df = 12, t = 5.72, p<10^−4^). In addition, non-decision times were longer in the accuracy condition (acc: 366±41 ms, spd: 323±37 ms; paired t-test, df = 12, t = 4.33, p<10^−3^). The increase of non-decision time is not necessarily expected. Ideally, non-decision time represents fixed delays in the system. However, it is possible that efferent non-decision times are somewhat longer in the accuracy condition if subjects were to respond more cautiously, and hence, with less vigor. Qualitatively, the observed effect can certainly be interpreted in this way, but the effect size (∼41 ms) seems large given the short distance that needs to be covered in order to press a button.

Despite the reasonable fit to the data, there was one systematic modeling error. The model predicted higher than observed accuracy in the speed-condition, and lower than observed accuracy in the accuracy condition. This effect is particularly visible for the neutral and incongruent stimuli (**Figure 6B&C**). Taken together, these results show that subjects were able to improve accuracy more effectively, i.e., by investing less extra RT, than the TN_22 model predicted. This implies that subjects can make use of an additional mechanism that allows them to trade speed for accuracy more effectively than predicted by only increasing the response threshold. We focused on three plausible mechanisms that could explain this unaccounted increase in accuracy: the onset mechanism as discussed above, and two alternatives: a novel mechanism based on the noise-floor of the integrator unit and a previously proposed mechanism that focuses on stimulus selection time [**17**].

### Threshold, Onset and Non-decision time model (TON_222)

The TON_222 model adds two degrees of freedom to the TN_22 model by allowing decision onset to vary as a function of speed-accuracy instruction. The resulting model has 6 degrees of freedom and provides a significantly better fit to the data (**Figure 6E–H**; Population-based Wilkes Chi-square test for log-likelihood of nested models, df = 26, *Χ^2^* = 490, p<10^−10^). For 12 out of 13 individual subjects the Wilkes Chi-squared test for log-likelihood in nested models is significant at a level of 0.01 or lower. As with the TN_22 model, we used the “leave-one-out” method to test if all 6 degrees of freedom are necessary. The accuracy of the fit was significantly reduced by dropping a degree of freedom from threshold (Wilkes Chi-square, df = 13, *Χ^2^ = 295*, p<10^−10^, individual subjects: p<0.01 for 12/13), decision onset (df = 13, *Χ^2^* = 212, p<10^−10^, individual subjects: p<0.01 for 9/13), as well as non-decision time (df = 13, *Χ^2^ = 83*, p<10^−10^, individual subjects: p<0.01 for 4/13).

We then tested whether speed-accuracy instructions have a systematic effect on decision-onset across all subjects by comparing the parameter estimates of decision onset in the speed and accuracy conditions. We found a significant delay of decision onset in the accuracy condition (acc: 30±29 ms, spd: −27±41 ms; paired t-test, df = 12, t = 5.4, p<10^−3^). Due to the right-skewed nature of the distribution (one outlier, see **Figure 6H**), median decision onset values may be more representative of true decision onset (acc: 39 ms; spd: −14 ms). Note that zero represents the time point at which stimulus selective information first reaches the integrator unit. Thus, when *t_0_*<0, decision onset occurs before any stimulus selective information is present i.e. the integrator processes noise until *t_0_*  =  0. In line with the TN_22 model we find a significantly higher response threshold in the accuracy condition (acc: 0.94±0.47 ms, spd: 0.63±0.37 ms; paired t-test, df = 12, t = 7.7, p<10^−5^). In contrast to the TN_22 model, the TON_222 model did not detect a significant increase of non-decision time in the accuracy condition (acc: 347±34 ms, spd: 342±28 ms; paired t-test, df = 12, t = 0.5, p = 0.6).

### Threshold, Onset, Non-decision time and Starting-point variability model (TONS_222X)

We further tested if it is necessary to include one (TONS_2221) or two (TONS_2222) additional degrees of freedom by allowing the starting-point variability of the integrator unit, *Var[X(t_0_)]*, to be a free parameter. Presumably, starting point variability determines the noise level of the integrator prior to stimulus onset. Hence, there is no a priori reason to believe that this should be affected by speed-accuracy instructions. However, it is possible that the noise level is unequal to zero (in contrast to what was assumed in all previous models). In addition, it is possible that attention is needed to maintain a low noise level in the integrator unit, and that speed-accuracy tradeoff is mediated in part by reducing this noise level. This assumption is particularly relevant, since decision onset may operate in a similar fashion: if decision onset is initiated too early (before stimulus-selective information hits the integrator), this would increase the noise level at the time at which stimulus-selective information reaches the integrator and deteriorates performance.

**Figure 7:**
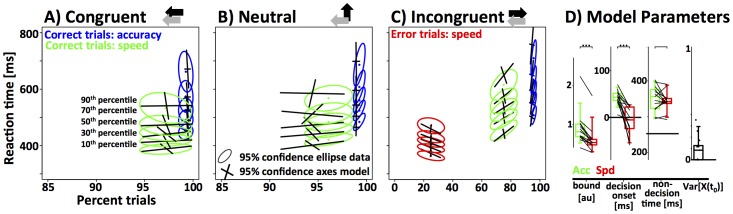
Fit of the threshold, onset, starting-point variability and non-decision time (TONS_2221) model to the data from the RT paradigm in (A–D).

The simulation results showed that the TONS_2221 model (**Figure 7**) provided a significant improvement over the TON_222 model (Wilkes Chi-square, df = 13, *Χ^2^* = 41, p<10^−4^, individual subjects: p<0.01 for 4/13). Further, the TONS_2222 model did not significantly improve the TONS_2221 model by adding another degree of freedom to starting-point variability (Wilkes Chi-square, df = 13, *Χ^2^* = 10 p = 67, individual subjects: p<0.01 for 1/13). At the same time, decision onset was still significantly different in the two speed-accuracy conditions (TONS_2221; acc: 42±16 ms, spd: −8±30 ms; paired t-test, df = 12, t = 5.6, p<10^−3^; TONS_2222; acc: 43±15 ms, spd: −6±31 ms; paired t-test, df = 12, t = 5.3, p<10^−3^). This indicates that the effect of decision onset is not mediated indirectly by affecting the noise level of the integrator at the time stimulus-selective attention reaches the integrator. Rather decision onset mediates its effect by reducing the impact of information from the salient but task-irrelevant distractor. In addition, it shows that subjects do not reduce the integrator noise level as a mechanism to increase accuracy.

The main difference between the parameters of the TON_222 and TONS_2221 model is decision onset in the speed condition. The TON_222 model produces an earlier decision onset, most likely due to the fact that an earlier decision onset is used to emulate starting-point variability in some subjects. After allowing an additional parameter for starting-point variability, decision onset became more homogenous across the population with values that were within a physiologically plausible range for all subjects. Unless mentioned otherwise, the TONS_2221 model is our preferred model and the basis for reporting parameter estimates of decision onset in the Discussion.

### Threshold, Non-decision time and Starting-point variability model (TNS_222)

**Figure 8:**
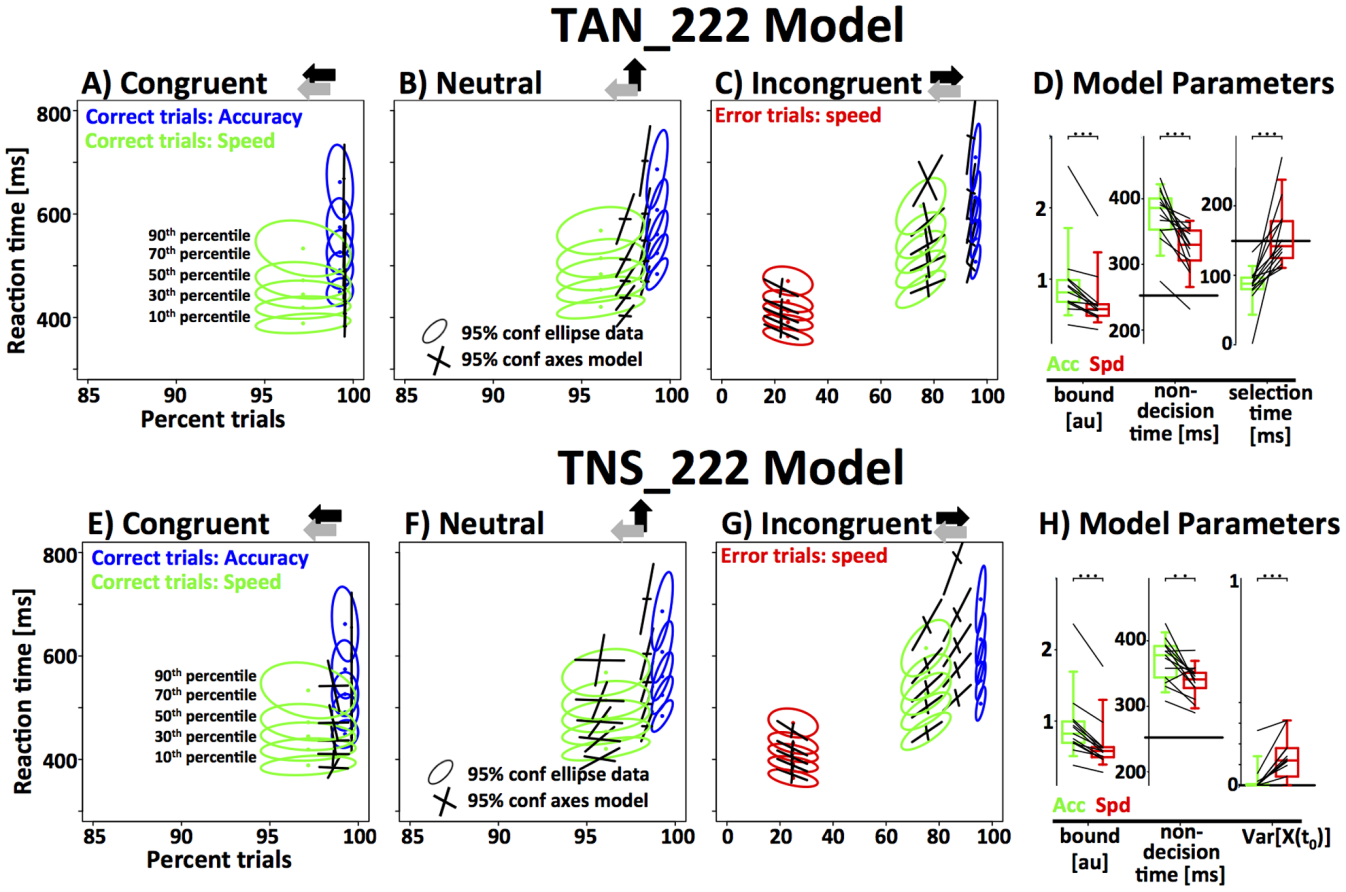
Fit of the threshold, selection-speed and non-decision time (TAN_222) model to the data from the RT paradigm in (A–D). (E–H) Fit of the threshold, non-decision time and starting-point variability model (TNS_222).

The previous analyses show that integrator noise is not necessary to model speed-accuracy tradeoff. However, the model used to test this assumption included decision onset. Hence, it is possible that the presence of decision onset prevented us from detecting an effect of integrator noise. To fully confirm the idea that integrator noise is not sufficient to explain speed-accuracy tradeoff, we constructed a model that drops decision onset completely and replaces it with staring-point variability (TNS_222, **Figure 8** **E–H**). As expected, the TNS_222 model allows a speed-accuracy tradeoff by increasing starting-point variability in the speed condition (acc: 0.027±16 ms, spd: 0.126±30 ms; paired t-test, df = 12, t = −4.43, p<10^−3^). Although the TNS_222 model provides a significant improvement over the TN_22 model (Wilkes Chi-square test, df = 26, *Χ^2^* = 188, p<10^−10^, individual subjects: p<0.01 for 5/13 subjects), it provides a less accurate fit than the TON_222 model (BIC TNS_222: 51,672; BIC TON_222: 51,067).

### Threshold, Attentional selection speed, and Non-decision time model (TAN_222)

Yeung and colleagues have presented an alternative account for speed-accuracy tradeoff in interference tasks [17]. The alternative account states that subjects increase top-down cognitive control in the accuracy condition. Higher cognitive control speeds up the stimulus selection process. Conversely, this leads to the counter-intuitive proposal that subjects increase response speed by deliberately slowing down the stimulus selection process. However, both accounts, the attentional selection speed and the decision onset account, improve accuracy in a very similar manner, namely by reducing the duration for which information from the salient distractor is affecting the decision process. The aim of the current simulation was to test whether one of the two accounts provides a numerically better fit to the data.

We emulated Yeung's model by allowing stimulus selection speed to vary between the two speed-accuracy instructions. The resulting model is referred to as the TAN_222 model. The selection speed model was designed to be as similar as possible to the TON_222 model. In particular, it contained the same number of free parameters (6). Four of those six parameters are shared between the two models: 2 degrees of freedom for non-decision time and 2 degrees of freedom for the response threshold. The remaining two degrees of freedom for decision onset were replaced with two degrees of freedom for stimulus selection speed.

**Figure 8A–D** shows the fits of the stimulus selection model to the data. Based on visual inspection, the model provides a reasonably good fit to the data. Because the two models are not nested, we could not use the standard Wilkes Chi-square test. Hence, we calculated an alternative metric, the Bayesian Information Criterion (BIC). Given that the two models have the same number of degrees of freedom, a comparison of the BIC values is very similar to the comparison of the underlying log-likelihood values. Based on the BIC comparison, the decision onset model provides a numerically better fit to the data (TAN_222: 51,350; TON_222: 51,067). It is also important to note that the TAN_222 model achieves the fit using very fast attentional selection times in the accuracy condition (acc: 84±29 ms, spd: 158±46 ms; paired t-test, df = 12, t = −4.8, p<10^−3^), a value substantially lower than that measured in the CD paradigm. Given that subjects had more training with the task by the time they perform the CD paradigm, it is surprising that they should be less effective at selecting the relevant target stimulus. Furthermore, the attentional selection speed account makes a second counterintuitive prediction – that selection speed should be faster in the condition with slower reaction times.

### “From-Scratch” models

One of the main strengths of our approach is that we leverage the CD paradigm to predict RT and accuracy in the RT paradigm. In this way, our estimate of the input to the decision unit (time-dependent drift rate, **Figure 4C**), is independent of our estimate of how the decision unit chooses to use this information (response threshold and decision onset). However, if for some reason, the CD paradigm provides erroneous estimates of drift rate, this could lead to erroneous conclusions from the RT paradigm. We thus tested whether the main effect of decision onset is dependent on the parameter estimates from the CD paradigm or whether the main effect could be replicated by allowing previously fixed parameters to be free parameters in the model. To that aim, we took the TON_221 model and allowed 5 parameters that were previously fixed to the values estimated from the CD paradigm to vary. In addition to the 5 degrees of freedom in the TON_221 model, the new model had another 5 degrees of freedom. We refer to this model as the CD_TON_221. To test if it is necessary to keep decision-onset in the model, we compared the CD_TON_221 model to a sub-model that did not include decision onset. This model is an equivalent extension of the TN_21 model, and will be referred to as CD_TN_21.

**Figure 9:**
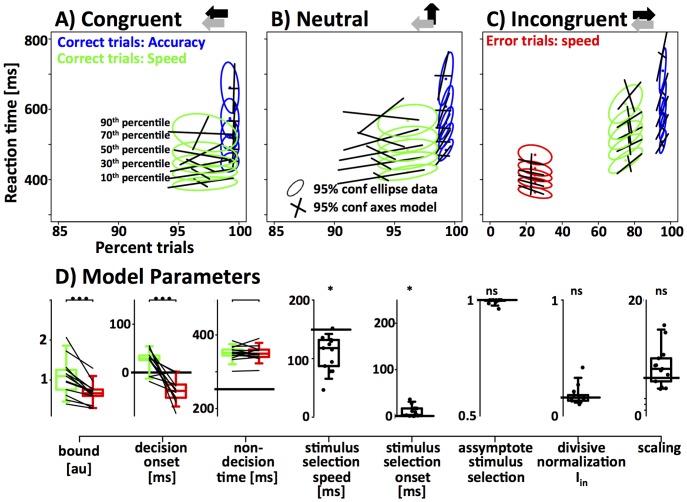
Fits of one of the “From-Scratch” models (CD_TON_222) to the data from the RT paradigm. Allowing the parameters of the CD model to vary provided a significant improvement to the fits. Three of the CD parameters were not significantly different from the values recovered from the CD paradigm. Two parameters (stimulus selection speed and stimulus selection onset) did show significant differences. Nevertheless, decision onset was needed to explain the speed-accuracy tradeoff.

The two new models allow us to answer two main questions: (1) can we confirm the role of decision onset for speed-accuracy tradeoff in this alternative analysis that does not depend on the CD paradigm, and (2) can we confirm the parameters that were estimated from the CD paradigm using the RT paradigm? To answer the first question, we compared the quality of the fits in the CD_TN_21 model with the fit of the CD_TON_221 model. Our results show that including the two degrees of freedom for decision onset provides a significant improvement to the fit (Wilkes Chi-square, df = 26, *Χ^2^* = 456, p<10^−10^, individual subjects: p<0.01 for 9/13). At the same time, decision onset was still significantly different in the two speed-accuracy conditions (decision onset in CD_TON_221; acc: 29±22 ms, spd: −31±30 ms; paired t-test, df = 12, t = 5.2, p<10^−3^). The same effect was present if we included an additional degree of freedom for non-decision time (**Figure 9**, decision onset in CD_TON_222; acc: 25±23 ms, spd: −36±30 ms; paired t-test, df = 12, t = 5.2, p<10^−3^). These analyses show that our main finding regarding decision onset is not specific to the drift-rates estimated from the CD paradigm.

In the next step we tested whether including the additional degrees of freedom for the CD parameters improved the fit. To that aim we compared the fit of the two CD_TON models with the fit of the two TON models. In both cases, we found a significant improvement of fit accuracy if the 5 additional CD parameters were included (TON_221 vs CD_TON_221:Wilkes Chi-square, df = 65, *Χ^2^* = 255, p<10^−10^, individual subjects: p<0.01 for 6/13; TON_222 vs CD_TON_222:Wilkes Chi-square, df = 65, *Χ^2^* = 256, p<10^−10^, individual subjects: p<0.01 for 7/13). We then compared the parameters estimated from the CD_TON_222 fit with the parameters estimated from the CD paradigm (**Figure 9D**). In particular, we were interested if the increased improvement of fit was achieved by systematic or random deviations from the parameters estimated from the CD paradigm. For two parameters, we found systematic changes: the onset and duration of the stimulus selection process (selection onset: one-sample t-test, df = 12, t = 2.8, p<0.05; duration of selection process: df = 12, t = −4.8, p<10^−3^). Based on the CD_TON_222 model, the stimulus selection process was estimated to begin ∼10 ms later, and be 40 ms shorter than estimated from the CD paradigm. Overall, the stimulus selection process was estimated to end approximately 120 ms after the first pulse of stimulus-selective information hit the integrator unit. Interestingly, this is the same number that was recovered from the CD paradigm if subjects 6 and 7 that exhibited the weakest timing performance were dropped from the sample. It is particularly important to note that even with the shorter attentional selection times of the CD_TON model, it was still necessary to allow decision onset to be a function of speed-accuracy instruction.

### Optimality of human decision strategies

**Figure 10:**
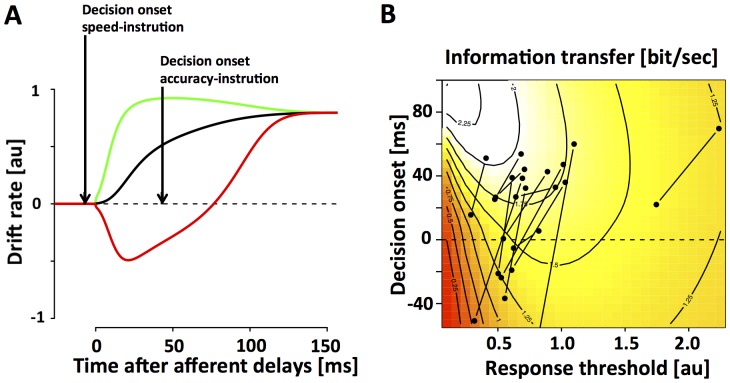
Parameter estimates for decision onset and response threshold. (**A**) Time dependent drift rate as estimated from the CD paradigm. The arrows indicate decision onset in the speed and accuracy condition as estimated from the TONS_2221 model. (**B**) Rate of information transfer as a function of response threshold on the x-axis and onset of the decision process on the y-axis. Overlaid are the parameter estimates for decision onset and response threshold from the TONS_2221 model. The lower left point on each line represents the parameters in the speed condition, the upper right point represents the parameters in the accuracy condition. All subjects delayed decision onset and increased response threshold in the accuracy condition. However, none of the subjects performed optimally. Information transfer in both conditions could have improved by further delaying the onset of the decision and lowering response threshold.

Based on our simulations, we concluded that the high rates of information transfer (>2 bit/sec) can be achieved by low response thresholds (<0.7) and late decision onset (>60 ms). The results of our simulations allowed us to test whether subjects picked optimal or close to optimal decision parameters. **Figure 10B** plots threshold and onset estimates from the TONS_2221 model on top of the map of information transfer. With the exception of one instance, none of the subjects reached high values >2 bit/sec. The majority of subjects increased the rate of information transfer in the accuracy condition. However, all subjects could have improved performance by using more optimal decision strategies: delaying decision onset and lowering response threshold. These adjustments could have been implemented without sacrificing the desired level of response latency or accuracy.

To estimate the contributions of the different mechanisms, we used the TONS_2221 model to predict changes in mean RT and accuracy if subjects had increased either decision threshold or delayed decision onset. The observed value was then normalized to the total value that was predicted based on the estimated adjustment of both response threshold and decision onset. Note that these analyses are approximations only and implicitly assumed a perfect fit of the model to the data. On average, the increase of decision threshold alone explained 73±16% (mean±std) of the increase in RT and 56±17% of the increase in accuracy. Decision onset alone explained 31±18% of the increase in RT and 75±23% of the increase in accuracy. Overall, decision onset was a much more effective way of trading speed for accuracy.

## Discussion

The current study examined the role of decision onset for optimal decision-making when selective feature-based attention is needed to reduce interference from salient but irrelevant distractors. Our study provides the first explicit measurements of how strongly feature-based selective attention biases the flow of sensory information on a millisecond time-scale over the time-course of a decision. Our results suggest that it takes ∼120 to 150 ms before the less salient but task-relevant target stimulus exclusively determines the input to the decision process. Based on the time-course of selective attention, we predicted that subjects could trade speed for accuracy by delaying decision onset, and that the onset mechanism could be more effective than the established threshold mechanism. The third part of our study shows that human subjects indeed use the onset mechanism to trade speed for accuracy: they automatically delay decision onset until selective attention can begin to isolate the relevant target when they are required to stress response accuracy over speed. The estimated delay of decision onset is substantial (50±32 ms), significant (p<10^−4^), and highly consistent across all subjects. On average, the observed 50 ms delay of decision onset alone would have accounted for an estimated 75% of the observed improvement in response accuracy while incurring only 31% of the cost in the form of longer response latencies.

### Alternative accounts: Attentional selection speed

It has been suggested that subjects stress accuracy by increasing cognitive control in order to minimize distractor interference, thereby accelerating the stimulus selection process [17]. In our case, this account would require that subjects were capable of accelerating the stimulus selection process *beyond* what was explicitly measured in the cyclic deadline task. This is unlikely because subjects were well-trained and motivated to perform optimally in all task conditions, especially the cyclic deadline version. If one were to assume that subjects were *not* performing optimally on the cyclic deadline task, such that greater cognitive control could and would have accelerated the selection process, then stimulus selection should be faster on trials following errors and incongruent stimuli, conditions believed to trigger cognitive control [17]. The lack of such an effect (Table 4 and 5) provides strong support for the argument that the speed of stimulus selection measured in the cyclic deadline task indeed forms a lower limit for the accuracy condition.

Alternatively, it is possible that the CD paradigm overestimated the time it takes to select the relevant stimulus. Two observations suggest that some overestimation could exist: (1) if the two subjects with the weakest overall timing performance (subjects 6 and 7, Tables 2 and 3) are excluded from the sample, stimulus selection times based on data collapsed across the remaining 5 subjects are reduced from ∼150 to ∼120 ms; (2) if the stimulus selection parameters are fit directly to the data from the RT paradigm, stimulus selection times are estimated to be ∼110 ms. In both cases, the **termination** of the stimulus selection process was estimated to occur 120 ms after stimulus onset. This is still significantly later than the 85 ms that are necessary to explain speed-accuracy tradeoff according to the attentional selection speed account (TAN_222 model).

A second argument against the attentional selection speed account is its somewhat un-intuitive main assumption: *greater* cognitive control and *faster* attentional selection result in *slower* responses in the accuracy condition, and *less* cognitive control and *slower* attentional selection result in *faster* responses in the speed condition. First, it is unclear why subjects would choose to invest less effort and cognitive control for the speed condition. After all, subjects are never instructed to use less effort in the speed condition; they are merely asked to stress a different aspect of the task. The speed-condition provides a very engaging experimental setting that should not go along with reduced effort. Second, faster attentional selection would be equally if not more helpful for the speed condition.

Overall, we believe that the onset mechanism provides a more intuitive account of the data: accuracy is increased by delaying the onset of the decision process to a more beneficial point in time; speed is emphasized by advancing decision onset to a less beneficial point in time. The subjects' motivation to do the best possible job in both conditions is reflected by the fact that the amount of cognitive control, and hence attentional selection speed, is assumed to be the same in both conditions. Because both mechanisms can mimic each other and provide similar overall effects in terms of accuracy and mean RT, it is important to note that fits achieved with the onset mechanisms (TON_222 model) are significantly better than the fits from the attentional selection speed account (TAN_222 model). Taken together, these considerations provide strong support for the delayed onset mechanism.

### Neural correlates of delayed decision onset

Our findings suggest that decisions are not necessarily initiated automatically by the presence of appropriate stimuli, but can be adjusted within limits to current task demands. The observed effects can only be explained by a neural mechanism that can delay the onset of the decision process without interfering with the allocation of selective attention. Note that this cannot be achieved by blocking out sensory input, for example, by closing one's eyes. In the absence of sensory input, selective attention cannot be allocated and the benefit of delaying decision onset is lost. To date we are not aware of a compelling neural correlate of delayed decision onset. Extra-cranial measurements of the onset of the lateralized readiness potential (**LRP**) are inconclusive because it is not clear whether LRP onset is more closely related to the onset of the decision process [11] or its termination [35]. Regardless of these problems of interpretation, different studies have either found effects of speed-accuracy tradeoff on LRP onset [35] or not [36], [37].

There is some evidence in favor of a gating mechanism from intracranial single-unit recordings [24], [38] in the frontal eye-fields (FEF). Based on the assumption that FEF *motor* neurons reflect the accumulation of sensory evidence that is represented in *visual* FEF neurons, these authors conclude that models with explicit gating of sensory evidence provide the best account of behavioral and neural data. Though the findings indicate the presence of a gating mechanism that delays the onset of the response selection process, the actual gating was not believed to occur in the temporal domain. Rather, the authors support a model with two nested firing rate thresholds, one that gates the flow of information from FEF sensory neurons to FEF motor neurons and a second threshold for response initiation.

A similar gating mechanism that operates in the firing rate domain might also play a role in our dot-interference task. However, there are strong arguments that favor temporal gating in our task. First, increasing the gating threshold for both motion channels would delay the integration of evidence for both channels, but this delay would not be equal for the two channels because the target contrast in our task was lower than the distractor contrast. Thus, it would take the target motion channel longer to cross threshold, thus increasing the impact of the distractor and partially reversing the desired effect. Secondly, in Purcell's case [24], the gating operates on the activity of visual FEF neurons that are believed to represent top-down mediated salience. These salience signals require significant preprocessing and have slow temporal dynamics that are reminiscent of neural integrators. In our case, however, the gating would be expected to operate on the activity of motion-selective neurons (e.g., in MT) that have much faster temporal dynamics and lack the slow increases of firing rate over time. Instead, MT neurons typically respond with a burst of activity to motion onset before they begin to become direction selective at a lower sustained firing rate. Because of this strong unselective burst of activity, firing rate dependent gating would affect decision onset only within a rather small range. Even more problematic, the predicted decision onset would always happen during the initial burst, i.e. before MT activity actually becomes selective for the direction of motion. The key finding of our study, however, states that subjects delay decision onset up to an average of 50 ms after the sensory evidence becomes selective for direction of motion (**Figure 10**). Despite these differences, we believe that there may be a way to partially reconcile activity-dependent and temporal gating as outlined below.

Our data indicate that decision onset can be affected by the instructions to the subject to stress either speed or accuracy. However, this is not necessarily the only determinant. In the context of the RT paradigm we find a potentially informative slight misfit of our model that may hint at an additional determinant of decision onset: RTs in the neutral condition are systematically longer than predicted by the model. In principle, this may be due to additional non-decision time that is specific to the neutral condition. However, the longer RTs also go along with higher than predicted accuracy, indicating that they might be due to a higher decision threshold or delayed decision onset (both mechanisms lead to longer reaction times and higher accuracy). But why would decision onset be delayed for the neutral relative to the congruent and incongruent condition? Here we speculate that decision onset may be modulated by the amount and/or salience of *potentially* task-relevant information. Potentially task-relevant information are dots moving either left- or rightwards. Dots moving up- or downwards are *a priori* irrelevant to the task. In the neutral condition, the distractors move upwards, thus reducing the amount of potentially task-relevant information. If our hypothesis is correct, this should delay decision onset, and increase RTs as well as accuracy in the neutral condition only. In summary, we speculate that that decision onset has at least two determinants: internal factors (speed-accuracy setting) and sensory drive in the shape of potentially relevant sensory information. This interpretation partially blurs the border between strict activity-based gating on the one hand (see above and [24]) and strict temporal gating on the other. Alternative explanations for the slight misfit in the neutral condition are possible, and at this point we have not conducted additional experiments to confirm our hypothesis.

### Subthalamic Nucleus and decision onset

In a number of groundbreaking recent studies, Frank and colleagues have dissected the role of the sub-thalamic nucleus (STN) for value-based decision making [39]. Their work has generated a neural network model of decision-making that is based on basal-ganglia anatomy and physiology (the BG model). In a recent paper, Ratcliff & Frank [40] set out to modify a numerically tractable diffusion model to capture the main properties of the more complex BG model. They argue that higher activity in the STN node of the BG model can be captured as an increase in decision threshold. The temporal dynamics of STN activity can be captured by exponentially collapsing response thresholds. Further, the authors suggest that STN activity can be so high as to prevent any responses from occurring and argue that this effect can be captured as an increase in non-decision time. To a certain extent our model shares common ideas with this body of work. In the following, we outline some of the most important differences.

(1) The model by Ratcliff & Frank contains one parameter, non-decision time, that represents both, the time of decision onset and conduction delays in the system. Our model, in contrast, splits this value into two independent parameters: decision onset and non-decision time. This distinction is not only possible, but also necessary, because in our paradigm drift-rate is non-stationary and varies systematically over the time-course of a single trial. As a result, we are able to distinguish whether subjects wait before initiating the decision process (delayed decision onset), or whether they wait to execute the response once they have made up their mind (longer non-decision time). Though Ratcliff and Frank present theoretical arguments that the former is the case, they provide no experimental evidence for the assumption that subjects delay decision onset, rather than increasing non-decision time. The two scenarios can only be distinguished experimentally when drift rate varies systematically over the time-course of a trial as is the case in our paradigm.

(2) Ratcliff and Frank propose that STN activity has two effects: an increase of the bound and an increase of non-decision time/decision onset. While the former is beneficial and helps subjects prolong the decision process in order to make more accurate choices, the delayed decision onset serves its purpose only if the quality of the sensory information improves over time. In our case, the delayed decision onset dramatically improves accuracy because it allows the decision to start at a point in time when the sensory information is more selective for the target stimulus. Hence, our study is the first demonstration that delaying decision onset can serve a purpose and improve response accuracy.

(3) In the Frank & Ratcliff model, STN activity, and hence decision onset, is determined by the amount of sensory conflict in the stimulus – that is, decision onset is not necessarily controlled by the subject. In our study, the stimuli are identical and the difference in decision onset must be mediated by the instructions. Hence, our study is the first demonstration that humans can actively manipulate decision onset to improve decision accuracy. Note, however, that subjects were not aware of how they were improving accuracy. Hence, most likely, decision onset is out of conscious control.

(4) Conversely, we do not allow our model to adjust decision onset as a function of sensory conflict. The main argument for this choice comes from the data of the CD paradigm that can be fit very nicely with a model that assumes identical decision onsets that are independent of conflict. However, the current study did not explicitly test whether the model would benefit from letting decision onset vary as a function of congruency. Hence, it is possible that future analyses may reveal such an effect.

Overall, we believe that our paper shares similarities with the work by Ratcliff & Frank. We would not be surprised if some of the differences outlined above could be attributed to differences in the tasks. In addition, we believe that the collapsing bound suggested by Ratcliff & Frank may increase the quality of the fits in our case, by reducing the over-dispersion that is observed for the RT quantiles in the neutral and incongruent condition.

### Challenges for the onset mechanism

A general challenge to the onset mechanism has been presented by Larsen & Bogacz [23]. They argue that an additional mechanism that determines decision onset would be computationally challenging and energy inefficient by adding an additional layer of complexity to the decision process. Here we have outlined that adjusting decision onset as a function of task demand (task set-dependent gating) is computationally no more demanding than adjusting response thresholds to task demands. The added complexity can be justified by the potential benefits of delaying decision onset that can far exceed those of response threshold adjustments. The benefit of delaying decision onset depends on the specific task in question. In artificial settings, when no distractors are present and momentary evidence is approximately constant over time, the potential benefit would certainly be smaller. In situations when targets and distractors are far enough apart, overt attention, i.e. a gaze-shift to the relevant location, may partially take over the role that covert allocation of attention plays in the current task [41]. However, in many realistic situations where relevant information is presented amidst very salient distractors, the effect of decision onset can be substantial.

### Sub-optimality of observed decision strategies

Our simulations highlight that our subjects set decision onset and response threshold at suboptimal levels — all subjects could have further decreased response latency without any loss in accuracy (**Figure 10**). To maximize the rate of information transfer, subjects would have needed to delay decision onset even further while lowering response threshold. This result raises the question: why did they not adopt a more optimal decision strategy? We will argue below that subjects use suboptimal strategies because they did not have enough time to find the optimal strategy. In the context of the threshold model, requirements of speed and accuracy are mutually exclusive and subjects can trade one for the other by changing threshold; that is, for any desired level of accuracy, there exists exactly one response threshold. The resulting response latencies are, thus, “*optimal*”.

Decision onset expands this one-dimensional view: the optimal strategy is best described as a two-dimensional vector that specifies response threshold and decision onset. Within this expanded framework, any desired level of accuracy or response latency can be achieved by an infinite number of threshold/onset combinations (iso-accuracy/iso-latency lines in **Figure 5A&B**). All settings will differ with respect to the rate of information transfer that they provide (**Figure 5C**) and the true challenge is not only to meet a required level of response accuracy/latency, but to do so while optimizing information transfer. Moreover, in this framework it may be imprecise to view the adjustment of speed and accuracy as a *tradeoff* rather than a two-dimensional *optimization* problem. This optimization problem is challenging given the stochastic nature of the dependent variables. For example, it will require hundreds of trials to reliably differentiate a decision strategy providing 99% accuracy from one providing 99.5%. A gradient-decent-like search for the optimal decision strategy would require tens of thousands of trials before converging on the optimal strategy. Hence, it is not surprising that the subjects in our task did not find the optimal strategy. It is possible, however, that subjects may learn to implement optimal decision strategies through additional training in combination with specific instructions that encourage them to delay decision onset and reduce response threshold.

### Comparison to other joint response signal/RT studies

Our study uses a powerful approach that includes two distinct experimental paradigms: a *response signal paradigm* and a *RT paradigm* with different requirements regarding speed and accuracy. This dual approach was pioneered by Ratcliff to study decision-making in a numerosity categorization task [33]. Here we applied the same general approach to study decision-making in the presence of salient distractors that cause stimulus and/or response conflict. Due to the different requirements of this type of decision, we adapted the methodology [33] in a number of ways, for example, by using the cyclic deadline paradigm instead of the standard response signal paradigm.

Early studies using a response signal paradigm assumed that information continues to accumulate until the response signal is given [30], [31]. Ratcliff [33], however, found that it was necessary to assume the presence of implicit response thresholds in the *response signal paradigm* in order to accurately predict data from the *RT paradigm*. Our study provides a somewhat different finding: the assumption of implicit response thresholds in the *response signal paradigm* (the CD paradigm) actually prevents accurate modeling of the data in the *CD paradigm itself*. The problem is that for very long processing times, subjects almost always respond accurately, even in the incongruent condition. This implies that the decision process never terminates at the threshold for the incorrect response. There are three ways to deal with this issue when modeling the data from the CD paradigm: (1) no response thresholds are present, (2) response thresholds are present but set to such extreme values that the incorrect threshold is never reached or (3) the response thresholds are not absorbing but reflecting, i.e. the integration of evidence continues even after the threshold has been reached. In the current paper we assume option (1) because it was conceptually, as well as computationally, the simplest alternative and provided a very good quantitative fit to the data in both paradigms. We believe that the apparent discrepancy with Ratcliff's earlier finding can be attributed to differences in the tasks (numerosity categorization vs. Stroop-like interference task).

### Perfect versus leaky integration

All modeling efforts reported here are based on the assumption of perfect integration. Perfect integration has been shown to be a good assumption in many different tasks and conditions. However, it is possible that a short integration time constant might be more beneficial in a task in which drift rate changes systematically over the time-course of a trial. In preliminary analyses we found that data from the CD paradigm can be explained quite well using perfect as well as leaky integration (data not shown). While it is possible that the RT paradigm may be able to distinguish between perfect and leaky integration, establishing this distinction was beyond the scope of the current paper. It is, however, worth mentioning that the only other model that predicts entire RT distributions in a similar interference task, also uses perfect integration [16].

### Comparison of non-decision times in CD and RT task

One surprising yet very consistent finding were the longer non-decision times in the RT paradigm (346±28 ms) compared to the CD paradigm (259±13 ms). In principle, it is possible that the novel CD paradigm did not provide accurate estimates of non-decision time. However, the non-decision times measured with the CD paradigm are in the expected range based on simple visual RTs [42] that were confirmed in an exploratory experiment using one example subject (data not shown). Overall, we argue that the non-decision times estimated from the CD paradigm seem to be a much more realistic estimate of the fixed delays in the system. This implies that (1) either non-decision times are not estimated correctly from the RT paradigm, or (2) non-decision times are actually longer in the RT paradigm. Option 1 is possible, in principle, but not very constructive because we cannot propose an alternative model that can explain the data using shorter non-decision times.

This leads to option 2. Here we consider 3 possibilities for why non-decision times could actually be longer in the RT paradigm: training effects, attention, and true paradigm-related differences. The simplest explanation of the lower non-decision times in the CD paradigm is additional training. By the time subjects performed the CD paradigm, they had performed several hundred trials in the RT paradigm, as well as a substantial amount of training in the CD paradigm itself. All of this additional exposure may have led to a more effective link between a stimulus/decision and the corresponding response. However, given the intuitive mapping between stimulus and response, we believe that the difference in non-decision time between the two tasks is larger than what would be expected from training effects.

It is also possible that these differences in non-decision time could be due to attentional effects. It is a well-known fact that attention can dramatically affect reaction times [42], in particular if a stimulus is presented unexpectedly. Hence, it could be argued that non-decision times are shorter in the CD paradigm because it provides a more engaging environment that makes it easier for subjects to focus attention on upcoming stimuli. In fact, based on our own observations and informal reports by the subjects, the CD paradigm was more fun and less tiring. However, it is also important to note that we took care to equalize temporal predictability of the stimulus: in both paradigms, subjects could anticipate stimulus onset. Hence, setting aside the ‘*fun-factor*’ of the rhythmic responses, there is no reason why subjects should not have been able to allocate the same amount of attention towards the time of stimulus onset in both paradigms.

Finally, it is possible that the two paradigms have inherently different non-decision times. Non-decision time in the RT paradigm may be longer because of additional post-decision overhead that is not present if subjects are pushed to their limit in the CD paradigm. It is unclear what this overhead would accomplish and why subjects would be willing to incur extra costs that do not prolong the decision process itself and hence do not contribute towards improving response accuracy. We speculate that the additional time might be used to initiate post-decision meta-analyses of the previous decision. Conversely, non-decision times might be shorter in the CD paradigm, because it allows subjects to influence their final motor response into a later stage of processing. If this were the case, we might expect to see more trials in which subjects accidentally press both buttons. Our data did not allow us to quantitatively test this assumption, but from our own subjective experience, this was not the case. If at all, double presses seemed to be more common in the RT paradigm. Regardless of what causes the longer non-decision times in the RT paradigm, our findings suggest that a cyclic response mode may be quite beneficial (by reducing non-decision times) in situations where subjects need to make many successive and potentially boring decisions.

## Conclusions

The current study provides support for the idea that subjects can strategically adjust decision onset to trade speed for accuracy in a Stroop-like interference task. The finding that decision onset can be adjusted to task demands has important implications for the development of training methods to help individuals make fast high-stake decisions in complex environments with salient distractors. Current strategies to improve accuracy suggest subjects should increase cognitive control in order to speed up the selection of the relevant target [17], [22]. While extensive training may produce some speeding of the selection process, there is certainly a physiological limit on how much the dynamics can be accelerated. Manipulating decision onset provides an independent mechanism to improve accuracy once that limit is reached by finding the optimal balance between decision onset and response threshold. Hence, our finding that decision onset can be controlled may spur the development of new training programs to delay decision onset, which could be particularly helpful for individuals or clinical populations that have a slow stimulus selection process or who make fast high-stakes decisions in complex realistic environments.

## Supporting Information

Figure S1 **The effect of biased competition on the activity of a single motion channel.** **(A)** Based on the temporal progression of the biased competition in Layer 2, activity of a motion channel can be grouped into two periods: the bottom-up period in which activity reflects physical salience, the top-down period in which activity reflects task-relevance. Note that in the temporal dynamics of the biased competition process were chosen for illustration purposes only and do not reflect the results of the fits to the actual data. Activity is plotted for six different stimulus configurations depending on the presence and direction of motion of the target and distractor dots (see Legend for color code). Conditions where either target and/or the distractor were not present serve as a reference, but never occurred in the actual experiment. Note that in the bottom-up period, the distractor alone (black line) elicits stronger activity than when it is present in combination with the target (green line). This reflects the finding that cells will respond with an average firing rate if two stimuli are presented in its receptive field simultaneously. Note that in the top-down phase, the green and cyan lines converge towards the grey line, while the orange line converges towards the red line. This means that the motion channel responds as if the distractor were less salient or even absent if the biased competition operates in a winner-take-all fashion. **(B)** The relative response strength of the individual conditions depends on the setting of the divisive inhibition term *I_in_* in equation (2) that models the non-linear contrast response function of the motion channel. All values represent activity in the bottom-up period. In the top-down period, responses converge to the grey, and red line, respectively, as indicated in (A).
(TIFF)

Figure S2 Data and model fits in the cyclic deadline task for all seven subjects separately. Conventions as in Figure 4.
(TIFF)

Figure S3 **RT fits for one example subject and example model (TON_222).** Predicted (green/red lines) and measured (black lines) RT distributions for one example subject in the RT paradigm. In different sessions, the subject was instructed to emphasize either speed (top row) or accuracy (bottom row). The central portions of the plot depict the density of the simulated decision variable as it develops over the time course of a trial (yellow: high density, red: low density, log-scale). The black line overlays accumulated mean drift rate as estimated from the cyclic deadline task. The insets above and below the central portion indicate the RT distribution for correct and error trials, respectively. The green and red lines indicate the fit of the 6-parameter TON_222 model to the data. For each condition, accuracy and RT distributions were fit with three free parameters: decision onset, t_0,_ response threshold, ±B, and non-decision time. All other parameters were set to the values estimated from the cyclic deadline version of the task.
(TIFF)
