# Supplementary figures and images for: Humans Optimize Decision-Making by Delaying Decision Onset
Source: PLoS One. 2014 Mar 5;9(3):e89638. doi: 10.1371/journal.pone.0089638 (PMC3943733; doi:10.1371/journal.pone.0089638)

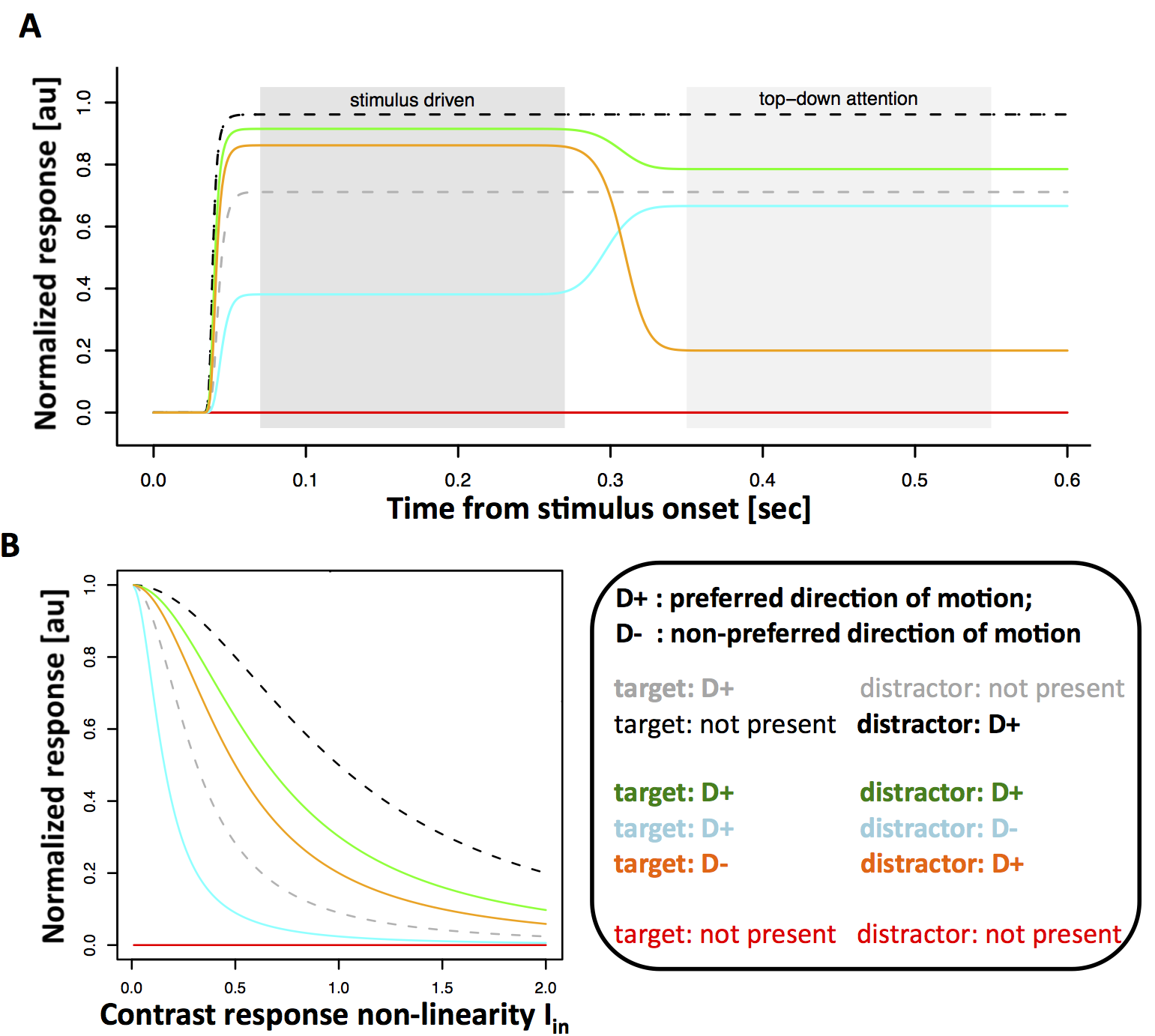

Supplement: Figure S1 — The effect of biased competition on the activity of a single motion channel. (A) Based on the temporal progression of the biased competition in Layer 2, activity of a motion channel can be grouped into two periods: the bottom-up period in which activity reflects physical salience, the top-down period in which activity reflects task-relevance. Note that in the temporal dynamics of the biased competition process were chosen for illustration purposes only and do not reflect the results of the fits to the actual data. Activity is plotted for six different stimulus configurations depending on the presence and direction of motion of the target and distractor dots (see Legend for color code). Conditions where either target and/or the distractor were not present serve as a reference, but never occurred in the actual experiment. Note that in the bottom-up period, the distractor alone (black line) elicits stronger activity than when it is present in combination with the target (green line). This reflects the finding that cells will respond with an average firing rate if two stimuli are presented in its receptive field simultaneously. Note that in the top-down phase, the green and cyan lines converge towards the grey line, while the orange line converges towards the red line. This means that the motion channel responds as if the distractor were less salient or even absent if the biased competition operates in a winner-take-all fashion. (B) The relative response strength of the individual conditions depends on the setting of the divisive inhibition term Iin in equation (2) that models the non-linear contrast response function of the motion channel. All values represent activity in the bottom-up period. In the top-down period, responses converge to the grey, and red line, respectively, as indicated in (A). (TIFF) [file pone.0089638.s001.tif]

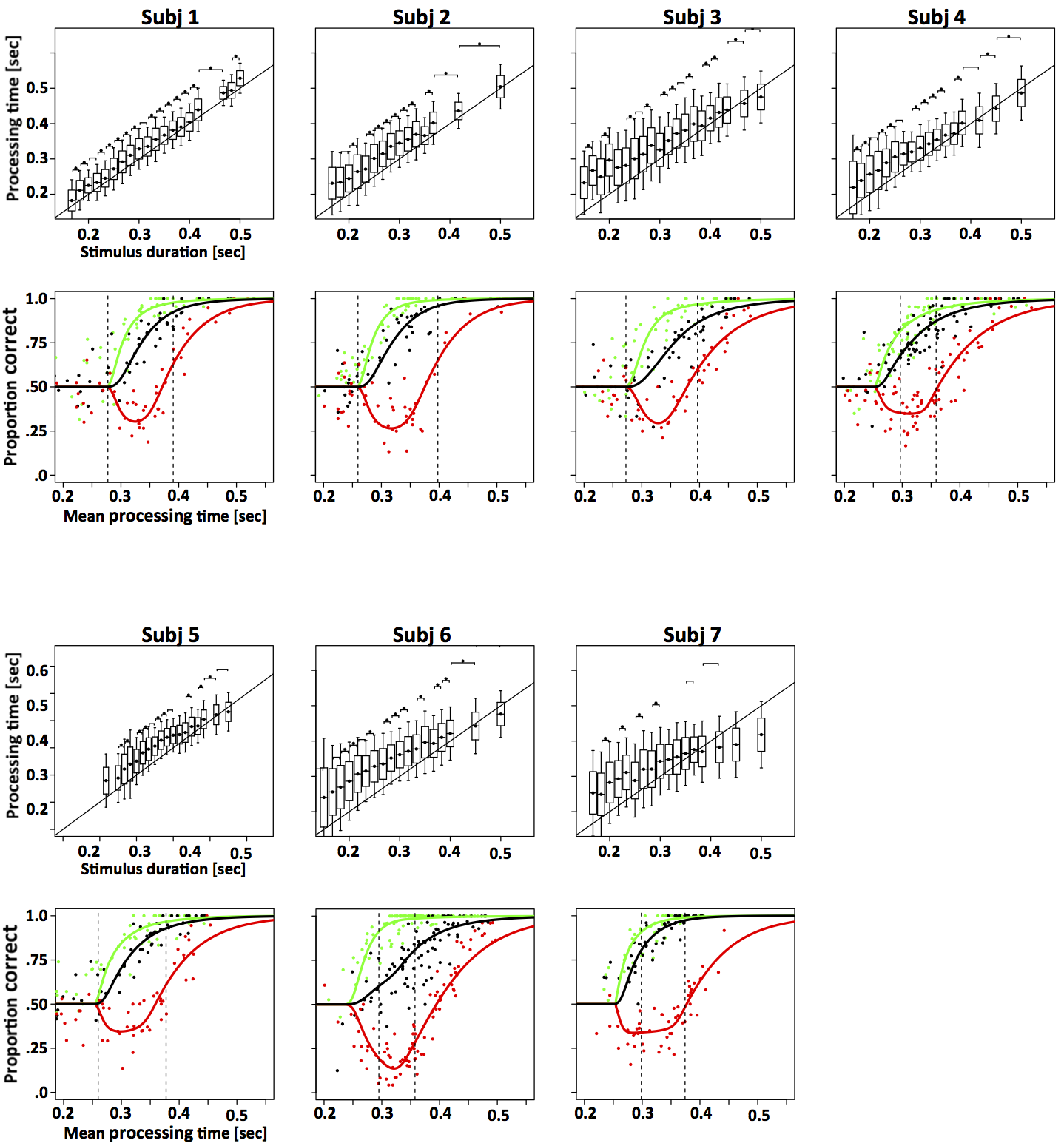

Supplement: Figure S2 — Data and model fits in the cyclic deadline task for all seven subjects separately. Conventions as in Figure 4. (TIFF) [file pone.0089638.s002.tif]

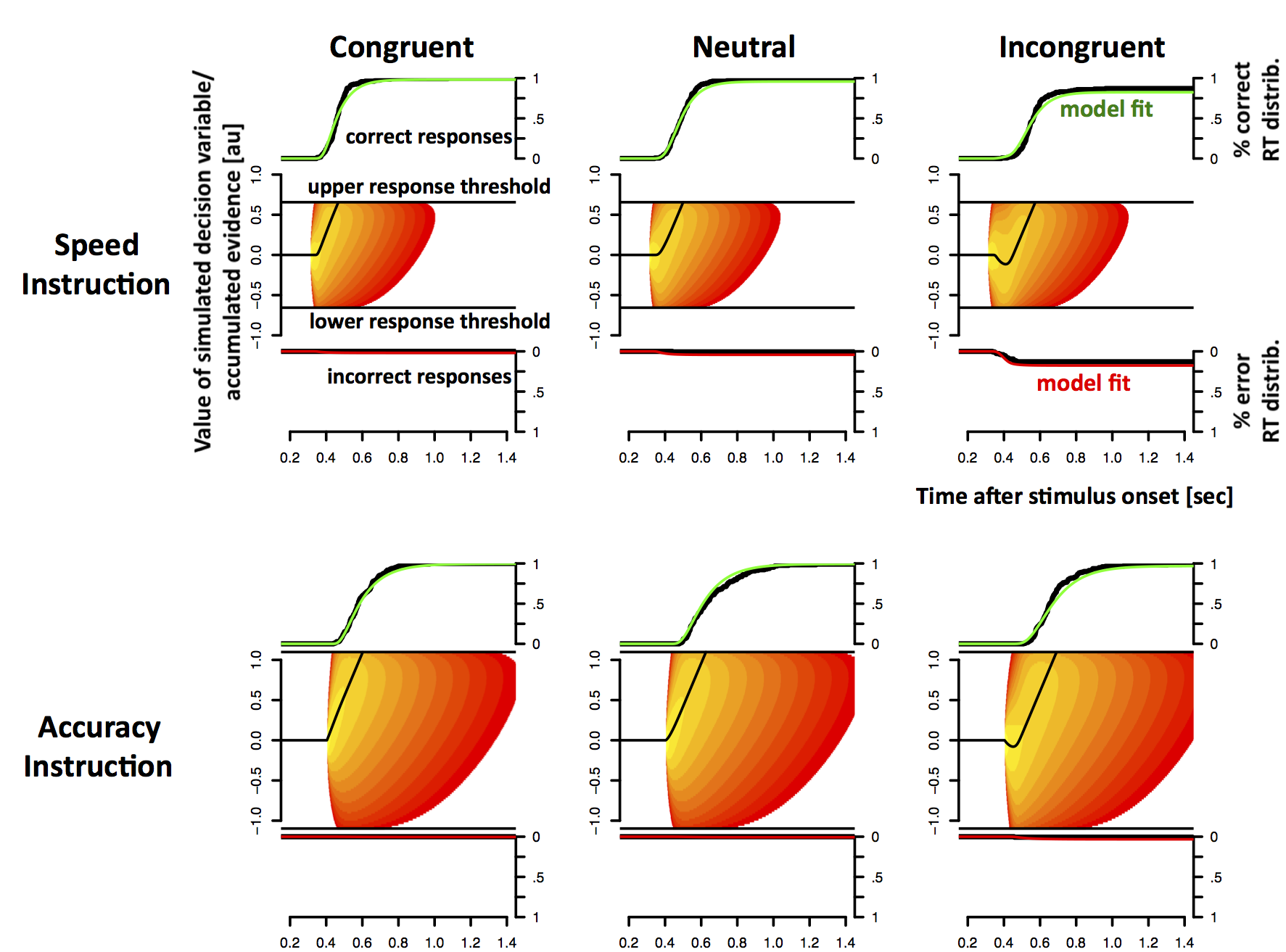

Supplement: Figure S3 — RT fits for one example subject and example model (TON_222). Predicted (green/red lines) and measured (black lines) RT distributions for one example subject in the RT paradigm. In different sessions, the subject was instructed to emphasize either speed (top row) or accuracy (bottom row). The central portions of the plot depict the density of the simulated decision variable as it develops over the time course of a trial (yellow: high density, red: low density, log-scale). The black line overlays accumulated mean drift rate as estimated from the cyclic deadline task. The insets above and below the central portion indicate the RT distribution for correct and error trials, respectively. The green and red lines indicate the fit of the 6-parameter TON_222 model to the data. For each condition, accuracy and RT distributions were fit with three free parameters: decision onset, t0, response threshold, ±B, and non-decision time. All other parameters were set to the values estimated from the cyclic deadline version of the task. (TIFF) [file pone.0089638.s003.tif]
